# Gene Targeted Therapies for Neurodegenerative Disorders: Strategies and Implications in ALS and SMA

**DOI:** 10.3390/genes17040419

**Published:** 2026-04-01

**Authors:** Ayse Yesbek Kaymaz, Gamze Bora-Akoğlu, Hayat Erdem Yurter, Christopher Grunseich

**Affiliations:** 1 National Institute of Neurological Disorders and Stroke, National Institutes of Health, 35 Convent Dr., Bethesda, MD 20892, USA; christopher.grunseich@nih.gov; 2 Faculty of Medicine, Department of Medical Biology, Hacettepe University, 06100 Ankara, Turkey; gamzeb@hacettepe.edu.tr (G.B.-A.); herdem@hacettepe.edu.tr (H.E.Y.)

**Keywords:** gene therapy, spinal muscular atrophy, amyotrophic lateral sclerosis, clinical trial readiness

## Abstract

Advances in technology have provided a better understanding of the genetic basis of neurodegenerative disorders and their underlying molecular pathophysiology. However, treating these disorders with conventional strategies is a major challenge. The approval of gene targeted therapy for spinal muscular atrophy (SMA) has laid the foundation for developing highly personalized therapies for other neurodegenerative disorders. As intensive research and efforts to advance gene targeted therapies continue, this review provides an overview of viral and non-viral vectors and delivery methods, as well as treatment strategies, including gene addition, replacement, editing, silencing, and splice modulation. Gene targeted approaches and clinical trials for SMA and amyotrophic lateral sclerosis (ALS) have demonstrated success, and additional studies are in progress. The design of efficient clinical trials which facilitate successful translation into clinical practice is of critical importance. Key considerations include the selection of appropriate disease models, understanding the natural history of the disease, and establishing well-defined outcome measures to assess prognosis of the disease and therapeutic efficacy. Finally, the precision of CRISPR-based gene editing offers the potential for one-time corrective therapies for monogenic disorders like SMA and SOD1-ALS.

## 1. Introduction

Neurodegenerative diseases (NDDs) refer to a group of disorders that cause progressive and irreversible loss of neurons in the nervous system. They affect millions of people worldwide and are considered one of the leading causes of death and disability [1]. The complexity of the nervous system and lack of relevant disease models have limited our understanding of the underlying disease pathophysiology in NDDs. Treating these disorders has always been a major challenge with conventional therapies due to the heterogeneous and complex nature of the nervous system, its slow regeneration capacity, the need for repeated dosing and the poor accessibility of the tissue due to the blood–brain barrier (BBB). As a result, there is no cure or disease-modifying therapy for many of these disorders. Nonetheless, advances in technology have provided a better understanding of the disease mechanism and subsequent advancement of gene targeted therapies for several NDDs.

**Figure 1:**
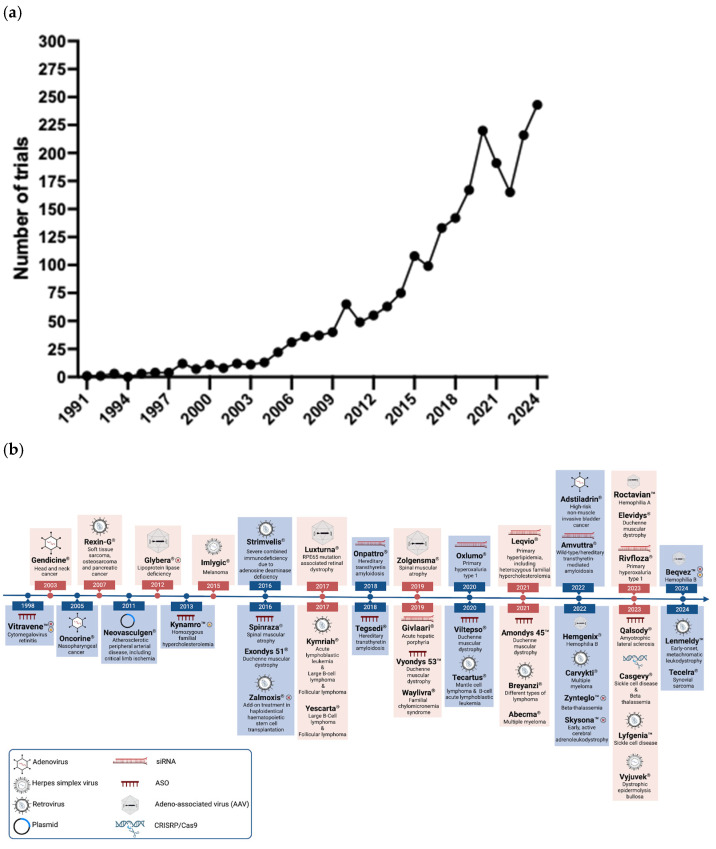
Gene therapy clinical trials and approved drugs. (**a**) Number of gene or cell therapy clinical trials that have been conducted in years between 1991 and 2025. The search was intended to provide a general overview of trends in gene- and cell-based therapy clinical trials over time rather than an exhaustive systematic analysis. Data were retrieved in 1 June 2025 from the WHO International Clinical Trials Registry Platform using keyword searches (e.g., ‘gene therapy’, ‘cell therapy’), and records were screened for relevance to gene targeted therapeutic approaches [3]. (**b**) Timeline of gene therapy drugs approved for clinical use. Note that not all drugs are approved in all countries. To illustrate advancements over time, drugs with withdrawn or expired authorizations, as well as discontinued therapies are also included. In the figure, these categories are marked with red (X) for Europe and orange (X) for the United States. Lentiviruses are not depicted independently of retroviruses. Sources include [4,5,6]. Created in BioRender.

Gene therapy is defined by U.S. Food and Drug Administration (FDA) as a technique that modifies a person’s genes to treat or cure disease by replacing a disease-causing gene with a healthy copy of the gene, inactivating a disease-causing gene that is not functioning properly or introducing a new or modified gene into the body to help treat a disease [2]. Gene targeted therapy studies have gained more interest due to advances in gene delivery and tools for gene manipulation, which has resulted in an acceleration of the current pipeline in recent years (Figure 1). To date, several gene therapy products have been approved, mostly for cancer. The pace of development for NDDs has increased, especially after the approval of the first gene targeted therapy for spinal muscular atrophy (SMA). Encouraging advances in personalized gene therapy studies have already been reported. Despite rapid advances in gene targeted therapies, a critical gap remains in their effective clinical translation. Considering the progress in the field, this review discusses gene targeted therapy strategies for neurodegenerative diseases by providing an overview of viral and non-viral vectors and delivery methods, as well as therapeutic approaches. Recent progress in SMA and amyotrophic lateral sclerosis (ALS) is highlighted, where gene targeted approaches and clinical trials have demonstrated promising outcomes. We further emphasize the importance and core elements of clinical trial readiness, which remain critical determinants of successful clinical translation, and discuss emerging strategies to improve the safety and efficacy of gene targeted therapies.

## 2. Delivery Methods, Vectors, and Strategies for Gene-Targeted Therapies

**Figure 2:**
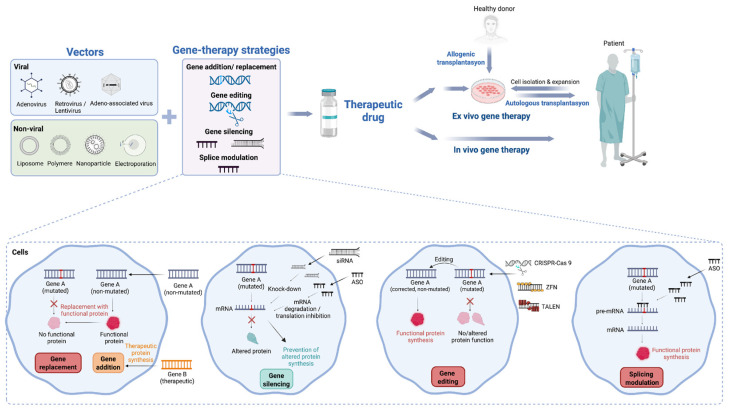
Vectors, delivery methods and gene-therapy strategies. Viral and non-viral vectors can be used in gene therapy strategies—including gene replacement, gene addition, gene silencing, gene editing, and splicing modulation—allowing the therapeutic drug to be delivered directly to the patient (in vivo) or administered after collection ofthe patient’s own cells (autologous) or donor cells (allogeneic) (ex vivo). siRNA, short interfering RNA; ASO, antisense oligonucleotide; ZFN, zinc finger nuclease; TALEN, transcription activator-like effector nuclease; CRISPR-Cas9, clustered regularly interspaced short palindromic repeats–CRISPR-associated protein 9. Created in BioRender.

In a broad sense, two therapeutic approaches can be implemented to introduce genetic material into the cells: in vivo and ex vivo (Figure 2). During in vivo gene therapy, genetic material is modified inside the body. With ex vivo gene therapy, cells are harvested from a patient (autologous) or a donor (allogenic) and then reintroduced to the patient after gene modification in culture. This approach is mostly implemented in blood-related disorders and is not suitable for many cell types that cannot be isolated from the body or that are not able to survive ex vivo for a long time [7,8]. Gene therapy’s progress to clinical reality has been accomplished by addressing vector choice and design, route of delivery, dosing and immune response of the patient and scalability. More research is needed to optimize the technology and extend its use for various monogenic or multifactorial disorders.

Delivery of therapeutic nucleic acid to the target site requires vectors to overcome a series of obstacles including the rapid clearance of genetic material by systemic endonucleases, a lack of tissue-specific distribution and a low efficiency of cellular uptake [9]. Immune system activation is another reason to choose a suitable delivery vector approach. Viral and non-viral vectors are used to deliver genetic material to the target cells or tissue. Each vector has advantages and disadvantages, and several points need to be considered when choosing a suitable gene delivery vector, including (1) the type and packing size of the genetic material, (2) the expression efficiency of the gene, (3) the duration of therapy, (4) the cytotoxicity and immunogenicity of the vector, (5) the feasibility of vector production, (6) the route of administration and targeted cell type, and (7) previous infection with the same virus chosen as a vector [10,11,12].

The majority of clinical trials (70%) have been conducted with viral vectors [6]. Viruses have long been used as gene delivery vectors for the treatment or prevention of neurodegenerative, muscular, cardiovascular, ophthalmological, and hematological diseases as well as cancer [13]. Adenovirus, adeno-associated virus (AAV), and retroviruses, including lentiviruses, are the most widely used vectors in clinical trials. Viral vectors are composed of three basic elements, including the transgene itself, the regulatory sequences that provide the expression and stability of the transgene such as the promoter, enhancer and poly-A regions, and the surrounding capsid [14]. Structural differences provide the opportunity for their preferred use in specific applications. Adenovirus (Ad) has a double-stranded linear DNA genome, containing early- and late-phase genes within “inverted terminal repeats” (ITRs), and is surrounded by a capsid. Different vectors are designed by deleting some or most of the genomic sequences to permit space for the transgene insertion. For instance, helper-dependent Ad vectors only contain ITRs and cis-packaging signals; therefore, they can accommodate 36 kb of exogenous DNA sequences [15]. Ad vectors have broad tropism and high transduction efficiency that enable infection of both dividing and quiescent cells. However, the presence of neutralizing antibodies from previous infections and the strong induction of immune response by Ads reduces their transduction efficiencies. To improve their clinical use, adenovirus serotypes can be genetically manipulated to reduce immunogenicity and increase transduction efficiency [14]. In 1965, a defective virus contaminant was identified in Ad preparations, which was later called AAV [16]. Today, AAVs are the main viral vectors preferred for in vivo gene therapy applications due to their broad tropisms, low immunogenicity, non-pathogenicity, rare genome integration potential and episomally durable transgene expression [13,17,18]. AAVs have a 4.7 kb single-stranded linear DNA genome, containing ITRs at both ends. Between ITRs, there are *cap* and *rep* genes for both replication and capsid synthesis. Vectors are designed by replacing both *cap* and *rep* genes with the transgene and tissue-/cell-specific or ubiquitous promoter, enhancer, 3′UTR region including regulatory elements and a poly-A tail [19]. There are at least twelve natural AAV serotypes, and hundreds of variants have been identified [13,17,20]. A specific AAV serotype can be selected to bind the desired target receptor and infect the cells of interest [13,20]. For example, AAV9, a clinically approved serotype vector delivering the survival of motor neuron 1 (*SMN1*) gene for SMA treatment, can infect any tissue including the brain by binding galactose as the primary receptor and laminin receptor 1 (LamR) and the AAV receptor as co-receptors [13]. After binding to their receptors, vectors are internalized via endocytosis and migrate through the trans-Golgi network towards the nucleus and escape endosomal vesicle entry before nuclear import. The capsid releases its single-stranded genome (ssDNA) in the nucleus and replication occurs to generate double-stranded DNA (dsDNA) that remains mostly episomal [21]. Nonetheless, a better understanding of the mechanisms of intracellular trafficking of different AAV serotypes would help to develop more specific and potent vectors [19]. In this regard, self-complementary AAVs (scAAVs) were generated by mutating one of the ITRs to by-pass the necessity of ssDNA to dsDNA conversion. As a result, transduction efficiency is increased, although the packaging capacity of the scAAVs is reduced to <2.5 kb from 4.7 kb [20]. scAAVs have successfully been translated into the clinic, as demonstrated using an scAAV9 vector in the treatment of SMA, which is the first neurological disease approved for AAV-based gene therapy. New vectors are designed to increase cell/tissue-type specificity, transduction efficiency, and reduce immune response via modification of either capsid or other elements, including the transgene cassette, promoter, enhancer or regulatory sequences [22].

In addition to AAVs, retroviruses are also used as vectors in the clinic for the treatment of different diseases. Retroviruses are enveloped viruses, having a single stranded RNA genome. Lentiviruses belong to the retroviridae family of retroviruses, capable of transducing both dividing and post-mitotic cells, including neurons. Lentiviruses have structural (*gag*, *pol* and *env*), regulatory (*tat* and *rev*), and auxiliary (*Vpu*, *Vpr*, *Vif*, *Nef*) genes in regions between long terminal repeats (LTRs) [14]. Vectors generated from lentiviruses lack required elements for viral replication and gene expression; therefore, they can deliver exogenous DNA almost 10 kb in size [23]. Vector tropism can be altered by modifying envelope proteins [24]. Due to their host genome-integrating ability, lentiviral and retroviral vectors are preferred for ex vivo gene therapy to provide long-term transgene expression, especially in hematopoietic stem cells and T cells. Currently, these vectors are used in CAR-T cell therapies for the treatment of blood cancers [13]. Lentiviral vectors prefer transcriptionally active sites to integrate, therefore increasing the risk of insertional mutagenesis. To overcome this issue, non-integrating lentiviral vectors have also been developed [13].

Although recent advancements in viral vector design and manufacturing have improved, several translational challenges remain to be addressed, starting with cytotoxicity and immunogenicity (including pre-existing immunity and inflammatory response) followed by limited genetic cargo capacity and the risk of off-target delivery or long-term safety concerns. Specifically, insertional mutagenesis is a phenomenon that raises concerns of malignancy, which can result from oncogene activation or tumor suppressor gene disruption during viral vector integration into the host genome [17]. For these reasons, non-viral vectors, therefore, have gained significant attention recently, after being ignored for years since they have poor delivery efficiency and transient transgene expression. Non-viral vectors have reduced cytotoxicity, immunogenicity and mutagenesis risk, flexible-insert-size DNA, cost-effectiveness, and ease of largescale production, and they have become an attractive area of gene therapy research [25,26].

The non-viral vectors are classified as naked DNA, as well as nanoparticle-based and chemical-based vectors. Small nucleic acid molecules (antisense oligonucleotides, ASOs; DNA interference, DNAi; RNA interference, RNAi; microRNA (miRNA) mimics; aptamers; and CpG oligodeoxynucleotides) and large nucleic acid molecules (plasmid DNA and mRNA) can be transferred with non-viral vectors by chemical and physical methods [25,27]. Chemical methods use synthetic or natural biodegradable particles such as inorganic particles, lipids, peptides and polymers to transfer nucleic acids to the cell. This delivery method is less destructive and can be used in vivo; however, it is less efficient than physical methods [28]. Physical methods include a variety of methods to introduce therapeutic nucleic acids into the cell. While needle injections and ballistic DNA injections introduce genetic materials directly into the cell or tissue, electrical pulse (electroporation), sound wave (sonoporation), hydrodynamics (hydroporation), laser pulse (photoporation) and magnetic fields (magnetofection) are used to permeabilize the cell membrane to allow nucleic acid entry [25,27]. Although high transfection efficiency can be achieved with physical methods, it has some drawbacks, including high cytotoxicity and difficulty of in vivo application. There are some challenges to overcome, including gene transfer efficiency, extracellular stability, internalization, intracellular trafficking, nuclear entry, specificity, gene expression duration, and safety [25,29]. To achieve efficient and safer gene targeted therapies, both viral and non-viral vector design and delivery methods need further investigation and improvement.

## 3. Gene Targeted Therapy Approaches for Neurodegenerative Diseases

Depending on the causative defect, gene targeted therapy strategies can include gene addition, gene replacement, gene editing, gene silencing and splice modulation therapies (Figure 2). These strategies can only be implemented in somatic cells as germline gene studies are not approved.

### 3.1. Gene Addition and Replacement

Understanding the genetic basis of diseases and the underlying molecular pathophysiology has facilitated the development of novel gene addition therapies. Gene addition strategies include the delivery of a functional copy of a faulty gene that could restore gene function in the context of monogenic disorders. In this case it is usually described as gene replacement. Furthermore, it is plausible to introduce a new gene targeting molecular mechanisms driving neurodegeneration or neuronal survival to compensate for the defective gene or to improve the disease phenotype. This approach is called gene overexpression and could be applied for monogenic diseases or disorders with complex etiology. Exogenous genetic material could be delivered locally or systemically via viral or non-viral vectors. The advantage of the gene addition approach is that the therapeutic protein can be continuously expressed in nondividing cells, potentially enabling one-time therapy. However, the use of viral vectors may be less effective in patients with pre-existing immunity to AAVs, which can reduce or eliminate therapeutic benefit. In addition, there is a potential of under- or over-expression of the therapeutic gene, and re-dosing is generally not feasible due to immune responses against the vector [30]. Furthermore, dose-dependent toxicity remains an important safety concern. Nonetheless, several challenges persist, including the need for efficient and safe delivery, minimization of off-target effects, and improved accessibility. While gene addition therapies have demonstrated substantial clinical benefit in certain disorders, including improvements in functional outcomes and survival, variability in response and long-term safety considerations highlight the need for continued optimization [30].

### 3.2. Gene Editing

Gene editing strategies allow for specific changes in the nucleotide sequence of the genome with the help of programmable nucleases [31]. Zinc finger nucleases (ZFNs) and transcription activator-like effector nucleases (TALENs) are the earliest gene editors preceding the CRISPR-Cas systems [32,33]. Two elements give ZFNs and TALENs the ability to modify specific regions of the genome: endonuclease Fok I and specially designed amino acids that guide the nuclease to the desired DNA domain. When the targeted DNA sequence is cleaved by Fok I nuclease, the endogenous dsDNA break repair mechanisms are activated, enabling gene disruption or precise alterations on this particular site [34,35]. Instead of synthetic peptides, the CRISPR-Cas system directs the nuclease domain to the targeted region by guide RNA sequences [36]. The CRISPR-Cas system is a natural adaptive immune system mechanism developed against bacteriophage infections and mobile genetic elements in bacteria and archaea [36,37,38]. The discovery of the potential of this system as a genome editing tool has resulted in unprecedented avenues for many research areas from disease modeling to gene therapy [36,39]. In principle, the guide RNA (gRNA) directs the Cas nuclease to the target genomic region, where the Cas protein introduces a double-stranded break in the DNA sequence complementary to the gRNA. Double-strand DNA (dsDNA) break repair activates either non-homologous end-joining (NHEJ) or homologous recombination (HR) repair mechanisms [40]. The error-prone NHEJ pathway causes frameshift or premature stop codon formation with insertions/deletions that occur during repair, providing a mechanism to generate loss of function in the target gene. In the HR mechanism, it is possible to repair the DNA precisely during mitosis—specifically in the S and G2 phases of the cell cycle when a sister chromatid is available as a template—and this high-fidelity repair can also be achieved when an exogenous template sequence is introduced. In this way, genome editing can be performed by providing a donor plasmid containing the desired nucleotide sequence (such as insertion, deletion, or point mutation). Over time, different versions of the CRISPR technique have emerged through the creation of Cas9 protein variants to overcome the limitations of dsDNA break dependence, efficiency and specificity issues. A catalytically inactive Cas9 protein (deadCas9 or dCas9) has been generated by engineering *Cas9* with nuclease-inactivating D10A and H840A mutations. Not able to create dsDNA breaks, dCas9 can be directed to the specific target sites to regulate gene expression at the transcriptional level enabling CRISPR interference and CRISPR activation [41,42]. Retaining only the D10A mutation, researchers generated the Cas9 nickase (nCas9) enzyme and fused the protein with a deaminase enzyme to create CRISPR base editors mediating precise, site-specific nucleotide conversions in the genome [39,43,44,45,46]. Prime editing is a more recent technology that was developed by fusing nCas9 (H840A) to a reverse transcriptase, allowing targeted insertions, deletions, and base substitutions with increased precision [45,47,48,49]. While base editors can perform a limited number of modifications to the genome, prime editing can introduce various types of modifications including base substitutions, insertions and deletions. These strategies are safer for neurons because they are post-mitotic and lack efficient HR repair, making DNA breaks especially harmful and prone to causing lasting damage and toxicity [50]. Continuous advancements in CRISPR-based platforms, extending beyond genome editing to include emerging RNA editing approaches, continue to improve precision and expand therapeutic potential for neurodegenerative disorders. Despite these advances, there are significant challenges of immune responses to Cas proteins, unintended genomic alterations, and the need for safe, efficient, and cell-type-specific delivery to the central nervous system (CNS) [50,51].

### 3.3. Gene Silencing

Gene silencing is used for the knockdown of a gene that is not functioning properly. Knockdown of gene expression can be achieved through oligonucleotide-based therapeutics, including gapmer ASOs, small interfering RNA (siRNA) or miRNA molecules. In recent years substantial progress has been made in the development of ASOs. ASOs are synthetic single-strand oligonucleotides that target mRNA molecules by sequence specific Watson–Crick base-pairing to cause either splice modulation or transcript knockdown [52]. Knocking down the mRNA transcripts can be accomplished by gapmers [53]. Gapmers are typically 15–22 base-pair long single-stranded ASOs that have a core DNA sequence complementary to target RNA (pre-mRNAs or mRNAs) and flanking sugar-modified RNA nucleotides at each end [54]. Gapmers are internalized by endocytosis and hybridize with RNA molecules in the nucleus or cytoplasm [55]. Formation of a DNA-RNA heteroduplex activates endonuclease ribonuclease RNaseH1 and induces cleavage of target RNA degradation, as well as release of the gapmer to bind to another target RNA [54,56,57]. siRNAs (20–25 base pairs) or miRNA mimics (~22 base pairs) are synthetic non-coding RNA molecules that can be used to regulate gene expression post-transcriptionally by inducing RNA interference. After introducing synthetic double-stranded siRNA and miRNA molecules, they are incorporated into the RNA-induced silencing complex (RISC). The sense strand of siRNA is degraded by argonaute 2 protein, while with miRNA it is unwound and released from the complex. The antisense strand guides the RISC to the complementary target mRNA, leading to either the cleavage of target mRNA or the interruption of translation. An siRNA perfectly matches one specific target mRNA and causes mRNA degradation, while a miRNA can target multiple genes at the same time and can be perfectly or partially complementary to the target mRNA. The degree of the miRNA complementarity to its target mRNA will determine the fate of the mRNA. Gapmer ASOs, siRNA and miRNA-mediated gene knockdown approaches can be considered a therapy for gain-of-function mutations if the resulting loss of function due to therapy causes no additional harm to the patient. This approach could also be useful for disorders caused by a dominant negative mechanism of action. Dominant-negative effect occurs when the mutant gene product interferes with the wild-type gene product. Knock down of the mutant gene expression could be a strategy to eliminate the dominant negative effect [58]. ASOs can be administered naked, whereas siRNAs and miRNAs require viral vectors or non-viral carriers for delivery [59,60,61]. To treat neurodegenerative disorders, oligonucleotide-based therapies must effectively reach the CNS; however, their major limitation is the inability to cross the blood–brain barrier due to their size and negative charge. As a result, delivery typically relies on intrathecal or intracerebroventricular administration, which have been performed safely but are invasive, may cause procedure-related inflammation, require repeated dosing, and can result in limited distribution to deep brain regions [62]. To overcome these challenges, several approaches are currently being developed to achieve effective and noninvasive delivery of oligonucleotides to the CNS. ASOs have been subjected to combinations of chemical modifications on the backbone (phosphorothioate bond) or sugar residues (e.g., 2′ -O-methyl, 2′-O-methoxyethyl, 2′-fluoro, locked nucleic acid and constrained ethyl modifications) to confer resistance to nucleases and to increase stability, binding affinity to RNA, tissue uptake, potency, solubility, and reduced toxicity [62,63,64,65,66,67]. siRNAs and miRNAs can have comparable chemical modifications to ASOs including modifications that would help to ensure correct loading onto the RISC, and efficient strand selection to unwind and discard the sense strand [68,69]. Recently developed divalent siRNAs, which are composed of two fully chemically modified, phosphorothioate-containing siRNAs connected by a linker, have shown promise for treating neurological disorders due to their potency, enhanced distribution in brain regions and long-lasting gene silencing effects with minimal toxicity [70]. Ongoing research in oligonucleotide chemistry and delivery methods holds promise for overcoming the current limitations of these therapies. Addressing manufacturing and cost challenges will be critical to improving accessibility for a larger patient population in the future. Nevertheless, several key questions remain, including how effective these therapies are in late-onset diseases or in conditions where early diagnosis is not possible, whether targeting the underlying cause in complex disease etiologies leads to meaningful clinical benefit, and whether these interventions remain effective when initiated after symptom onset [62].

### 3.4. Splice Modulating

In addition to their degrading mechanisms, ASOs can act as steric blockers to upregulate or downregulate gene expression [71]. Splice modulating ASOs can alter pre-mRNA splicing by binding to splice sites, enhancers, or silencer sequences and result in exon skipping or inclusion [72]. In addition, they can alter mRNA stability by changing polyadenylation site selection and inhibit translation by sterically blocking ribosomal subunits and RNA binding proteins [73]. Also, they can increase the translation of a protein from its main open reading frame (ORF) by blocking the upstream open reading frame (uORF) within the 5′UTR [64,74,75]. These mechanisms allow splice modulation therapies to restore the reading frame to increase protein levels or modify the transcript to synthesize a partially functional protein where the disease mechanism is loss of function [58]. By masking splicing regulatory elements from the spliceosome machinery, they can also disrupt reading frames to decrease protein levels when the disease mechanism is from a gain of function. In the case of haploinsufficiency or dominant negative effect, upregulating the expression of the wild-type allele is also possible [74]. The flexibility and established safety of splice-switching ASOs in neurological disorders, together with advances in transcriptomic and computational approaches, make them a powerful strategy for targeting previously untreatable neurological conditions and rare mutations [71]. While variant- and exon-specific splice-modulating ASOs enable highly individualized treatments, gene-focused approaches can target a broader range of patients with diverse mutations within the same gene, expanding therapeutic reach [71]. Additional new ASO strategies will be emerging as our understanding of ASO and disease pathomechanisms continue to expand [58].

## 4. Current Gene Targeted Therapies in Neurodegenerative Disorders: SMA and ALS

### 4.1. Spinal Muscular Atrophy (SMA)

SMA is a rare inherited disease primarily affecting children. *SMN1* gene mutations, especially deletions, are responsible for SMA. Almost 95% of the patients have homozygous deletions in exon 7 or both exons 7 and 8; however, the clinical phenotype is substantially different among patients who have the same mutation. Historically, SMA is classified into five groups according to the age of disease onset and achieved motor functions. Type 0 is the most severe form of the disease, which begins prenatally and survival is generally few days to weeks. Alternatively, symptoms can occur after the age of 30 in type 4 patients with a normal life expectancy [76]. *SMN1* is not the sole gene that encodes survival motor neuron (SMN) protein in the human genome. The nearly identical copy of *SMN1*, namely *SMN2*, exists in multiple copies ranging from 1 to 6. However, due to the C to T transition, splicing of *SMN2*-encoded mRNAs is defective, and almost 90% of transcripts lack exon 7. Therefore, a minor fraction of the transcripts are available for the translation of full-length and functional SMN protein. The increased copy number of *SMN2* allows for the synthesis of more full-length SMN protein; therefore, higher *SMN2* copy numbers are associated with milder phenotypes. Due to the inequivalence of *SMN2* copies—caused by variations within the gene and partial conversions between SMN genes—clinical severity does not absolutely correlate with *SMN2* copy numbers [77,78]. *SMN2* is not the sole modifier of the SMA phenotype; the effects of other genes, such as plastin 3 and NCALD, have also been demonstrated [79,80]. However, *SMN2* is the primary target of two of the three approved treatments for SMA.

**Figure 3:**
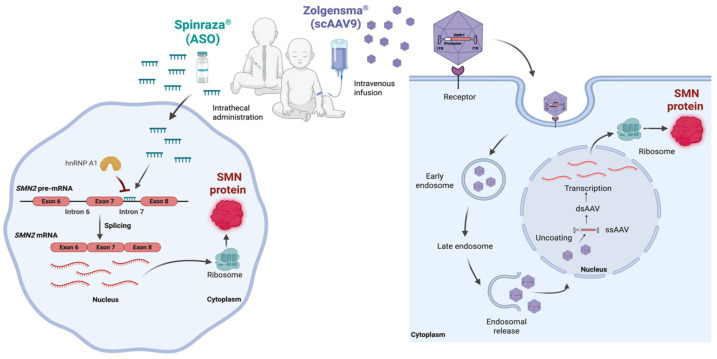
Mechanisms of clinically approved therapies for SMA. Spinraza and Zolgensma, used in the treatment of SMA, differ in their mechanisms of action. Spinraza induces exon 7 inclusion in *SMN2*-encoded mRNA by preventing the binding of hnRNP A1. Zolgensma enables the synthesis of functional SMN protein through *SMN1* gene replacement using the scAAV9 vector. SMA, spinal muscular atrophy; SMN, survival motor neuron; hnRNP A1, heterogeneous nuclear ribonucleoprotein A1; scAAV9, self-complementary adeno-associated virus serotype 9; ITR, inverted terminal repeat; ASO, antisense oligonucleotide. Created in BioRender.

Deficiency of the SMN causes several cellular perturbations that lead to the progressive degeneration of alpha motor neurons in the spinal cord and results in symmetrical muscle weakness and atrophy. Moreover, non-neuronal tissues, including skeletal and cardiac muscle and kidney, have also been reported to be affected by the loss of function, and SMA is considered a multisystem disease [81,82,83]. SMN functions in different cellular processes, including snRNA biogenesis, endocytosis, translation, and cytoskeleton regulation; therefore, loss of SMN impairs multiple molecular mechanisms that are currently under investigation [84,85,86,87,88,89]. Restoration of SMN-related perturbations by enhancing SMN protein level have been extensively studied in both pre-clinical and clinical studies [90,91,92]. At the end of these efforts, the first success came from studies with an ASO, namely Nusinersen (Spinraza^®^). Nusinersen was approved in 2016 by the FDA and then by the EMA and is now currently available for the treatment of all types of SMA. Nusinersen is delivered intrathecally to reach motor neurons, which are the most sensitive cell types affected by SMN deficiency. The ASO targets *SMN2*-encoded pre-mRNAs, aiming to restore the inclusion of exon 7. Nusinersen is an 18-mer long oligonucleotide, which has backbone modifications of 2′-O-2-methoxyethyl phosphorothioate to provide protection from nucleases [93]. The ASO enters the nucleus and binds to the intronic splice silencer N1 (ISS-N1) sequence, located in the immediate downstream 5′ splice site of the intron 7 *SMN2*-encoded mRNA. Thereby, it prevents the binding of heterogeneous nuclear ribonucleoprotein A1 (hnRNP A1) and leads to the inclusion of exon 7 (Figure 3). Nusinersen has been used in more than 14,000 SMA patients to enhance cellular SMN protein levels [93,94,95,96].

In 2019, a second therapeutic approach also gained approval from the FDA, namely onasemnogene abeparvovec (Zolgensma^®^), and more than 4000 SMA patients have received this therapy [97]. Onasemnogene abeparvovec is a gene replacement therapy, aiming to enhance SMN protein level by AAV9-mediated *SMN1* gene delivery. SMN cDNA, together with the human cytomegalovirus enhancer and chicken beta actin promoter, is packed into a non-replicating scAAV9 capsid. The journey of AAV vectors starts with cellular internalization via receptor-mediated endocytosis. After release from the endosome, it passes through the nuclear pores and is uncoated. In the nucleus, single-stranded DNA forms a double strand via complementarity at inverted terminal repeats, thus allowing for transcription to occur. mRNAs leave the nucleus, and the target protein is translated in the cytoplasm [13,92]. Onasemnogene abeparvovec can increase the level of functional SMN protein in both neuronal and peripheral tissues due to its distribution by intravenous administration [98].

To correct *SMN2* splicing, small molecules have also been studied extensively. The approval for SMA therapy came with risdiplam (Evrysdi^®^) in 2020, and its tablet form was subsequently approved in 2025 [90,91,99]. Several analogs have been shown to modify *SMN2* splicing in pre-clinical studies; in fact, the mechanism of action has been demonstrated for its analogues but not for risdiplam itself at the time. It has been reported that the SMN-C3 analogue interacts with the AG-rich motif within exon 7, while analogue SMN-C5 stabilizes U1 snRNP on the 5′ splice site. A recent in vitro study with risdiplam demonstrated the effects of an AC-containing motif in exon 7 on exon inclusion and proposed a mechanism for its mechanism of action [100]. The advantage of risdiplam over nusinersen is oral delivery, thereby reaching not only the nervous system but also peripheral tissues. However, off-target effects are the major drawbacks that have been recently reported in vitro [93,100,101,102]. The effects of risdiplam on patients previously treated with the aforementioned therapies have been recently reported [103]. The effects of risdiplam in combination with onasemnogene abeparvovec, as well as switching from nusinersen to risdiplam, have been investigated [104,105].

Translational medicine has helped to develop therapies for SMA, starting from the bench to the bedside. However, this is not the end, since further clinical and molecular investigations are needed to understand the effects of these therapies on cellular mechanisms and implications for improving patient health. Clinical studies of ASOs (NCT05067790-Phase IIIb, active, not recruiting- for a higher dose of nusinersen on patients who were treated with risdiplam; NCT05575011-Phase I, active, not recruiting- for another ASO, BIIB115) and gene therapy (NCT05335876-Phase III, recruiting for OAV101) approaches are ongoing for SMA [106,107].

### 4.2. ALS

ALS is a fatal neurodegenerative disorder that results from progressive motor neuron degeneration in the brain, brainstem, and spinal cord [108]. Resulting muscle weakness and paralysis lead to respiratory failure and death within 2–4 years from diagnosis [109,110]. The worldwide incidence of ALS is 2/100,000, and the mean age of adult-onset ALS varies between 40 and 63 years [111,112,113].

ALS is classified either as familial (fALS; 10% of the cases) or sporadic (sALS; 90% of the cases); however, this classification overlooks the complex genetics that underlie ALS pathophysiology [114]. More than 40 genes have been associated with the disease and known gene mutations can explain 70% of the fALS and 15% of the sporadic cases [114,115,116]. The four most common ALS-related genes are superoxide dismutase 1 (*SOD1*; MIM147450), fused in sarcoma (*FUS*; MIM137070), TAR-DNA binding protein (*TARDBP*; MIM605078), and chromosome 9 open reading frame 72 (*C9orf72*; MIM614260). These four genes account for 40–55% of fALS and 5% of sporadic cases and have different frequency, inheritance pattern, and penetrance. The disease pathophysiology is complicated and incompletely understood [114,117,118]. Consequently, ALS treatment has been limited to a small number of drugs approved at the time of this review: Riluzole (1996 EMA and 1995 FDA approval), Edaravone (2017 FDA approval) and Relyvrio (2023 FDA approval). These drugs do not have specific targets, their exact mechanism of action is not fully understood, and they have a modest effect on the disease phenotype. Tofersen (Qalsody^®^) was the first precision medicine approved for *SOD1*-ALS by the FDA in 2023. This highlights the urgent need to develop more targeted and effective therapies, leveraging advances in genetic testing and gene-based treatments.

As gene targeted therapies are challenging for various neurological disorders, the field of ALS therapeutics has its own challenges due to the complex etiology of the disease and the need for effective delivery to both the cerebral cortex and the anterior horn cells of the spinal cord [59]. Since toxic gain of function is the predominant mechanism in ALS, several approaches reaching clinical trials have been implemented to silence gene expression and treat the genetic cause of the disease. Current clinical studies of the gene targeted therapies in ALS are summarized in Table 1.

#### 4.2.1. SOD1

**Figure 4:**
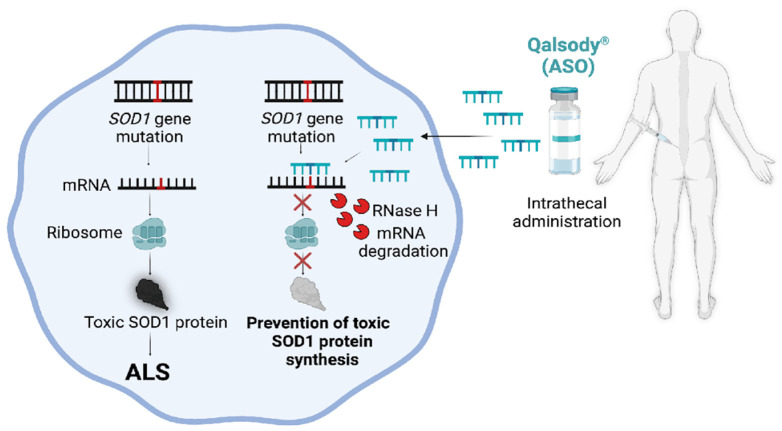
Mechanisms of clinically approved ASO-based therapy for ALS. Qalsody^®^ (Tofersen) prevents the aggregation of toxic SOD1 protein via inducing mRNA degradation by RNase H. ALS, amyotrophic lateral sclerosis; *SOD1*, superoxide dismutase 1; RNase H, ribonuclease H; ASO, antisense oligonucleotide. Created in BioRender.

More than 200 mutations have been reported in the *SOD1* gene since its discovery in 1993 as the first gene associated with fALS [117]. *SOD1* mutations account for 2% of all ALS cases (10–14% of fALS and 1–2% of sALS in European ancestries) [117,120]. The *SOD1* gene encodes the superoxide dismutase enzyme, which is a metalloprotease that metabolizes superoxide radicals to molecular oxygen and hydrogen peroxide, thus providing a defense against oxygen toxicity [121,122,123]. Although the underlying disease pathology is not fully understood, the predominant mechanism is the gain of function, with rare loss-of-function studies also reported [124]. Lowering the concentration of misfolded and aggregated mutant SOD1 protein offers a potential strategy for therapy, with gene editing, RNAi, and ASO-based approaches implemented to silence mutant *SOD1* gene expression [125,126]. ASO 333611 is the first promising candidate to treat SOD1-ALS; it was able to decrease both SOD1 mRNA and protein levels in rats and extended survival by 37% after disease onset [127]. These encouraging results have led to a groundbreaking first-in-human clinical trial (NCT01041222), showing, for the first time, the feasibility of the intrathecal administration of CNS-targeted ASOs to treat genetic forms of ALS [128]. Advancements in ASO technology have facilitated the development of a more potent ASO called Tofersen (BIIB067) [129]. Tofersen (Qalsody^®^) is the first FDA-approved precision medicine for a genetic form of ALS (Figure 4). The molecular formula of Tofersen is C_230_H_317_N_72_O_123_P_19_S_15_, which contain 20 bases with an RNA–DNA–RNA (5–10–5) gapmer mixed backbone containing an oligonucleotide that has a molecular weight of 7127.86 atomic mass units [130]. After the entry to the motor neurons and astrocytes, Tofersen forms a DNA:RNA hybrid inside the cytoplasm, which is recognized and cleaved by the enzyme RNaseH1. In rodents and non-human primates, Tofersen was able to decrease *SOD1* mRNA and protein levels, significantly extending survival, and decrease serum and cerebrospinal fluid (CSF) plasma neurofilament light chain (NfL), which are biomarkers of axonal injury and neurodegeneration. Tofersen enabled the reversal of neurodegeneration even after disease onset [129]. ALS patients with *SOD1* mutations received ascending doses of Tofersen by intrathecal administration over a period of 12 weeks in phase I/II [131]. The safety and efficacy of Tofersen was then evaluated in a phase 3, randomized, double-blind, placebo-controlled VALOR trial (NCT02623699) and a long-term extension study (NCT03070119, completed) [132]. Procedure associated adverse events were common and neurological adverse events were observed in approximately 7% of patients. The primary endpoint of the trial was changed from baseline to week 28 in the Amyotrophic Lateral Sclerosis Functional Rating Scale-Revised (ALSFRS-R) total score in the faster-progression subgroup. Although Tofersen did not meet the clinical end points, the drug was able to reduce CSF SOD1 protein levels and plasma concentrations of NfL in patients [132,133]. NfL levels were considered a biomarker that can reflect possible benefit, and based on this, Tofersen was approved by the FDA in April 2023 and an expanded access program is ongoing (NCT04972487).

Another approach to lower SOD1 protein involves RNAi molecules. ARO-SOD1 (siRNA), RAG-17 (siRNA) and AMT-162 (also known as. APB-102; adeno-associated virus rh10 containing an anti- *SOD1* miRNA) have recently been developed to treat adult patients with *SOD1* mutations [126,134,135,136]. The preclinical studies in rodents and non-human primates have shown improved efficacy, a long duration of action, and potency, as they delayed disease progression, preserved motor function, and extended survival. A study in two SOD1-ALS patients with A4V and D90A mutations showed the safety of intrathecal infusion of adeno-associated virus rh10 containing an anti- *SOD1* microRNA as a potential treatment. This proof-of-concept study lead the way for the phase I/II clinical trial of AMT-162 (NCT06100276), which recently enrolled 20 SOD1-ALS patients. A first-in-human trial of RAG-17 (NCT05903690) showed safety and efficacy in SOD1-ALSpatients [137]. CSF SOD1 and plasma NfL levels were significantly decreased. Moreover, the ALSFRS-R score was improved for all patients, and forced vital capacity was stabilized. These results show promise for treating SOD1-ALS patients with RAG-17 (NCT06556394).

#### 4.2.2. FUS

Discovered in 2009, *FUS* gene mutations are known to cause a rare-early onset, rapidly progressive form of ALS (ALS6) that is responsible for 4% of familial and 2% of sporadic cases [138,139,140,141]. The vast majority of the mutations are inherited in an autosomal-dominant fashion and mostly impact the C-terminal nuclear localization signal (NLS) region of the protein [142]. As a result, FUS protein is mislocalized to the cytoplasm and forms cytoplasmic inclusions. Consequently, underlying disease pathology is suggested to be both loss of function in the nucleus and gain of toxic function in the cytoplasm. A potential approach for therapy involves reducing levels of mutant FUS protein [143,144]. Jacifusen (also referred to as ION363 or Ulefnersen^TM^) is an ASO molecule that targets the *FUS* gene and reduces FUS protein levels [144]. Jacifusen was first introduced to a 25-year-old patient (JH) with an *FUS* P525L mutation whose identical twin sister passed away from the same mutation [144,145]. Although jacifusen was well-tolerated and slowed the rate of ALSFRS-R score decline, she passed away almost a year after starting treatment. The autopsy examination has revealed that jacifusen has a broad distribution throughout the CNS, even 2 months after the last therapeutic infusion, and dramatically decreased mutant *FUS* expression and FUS-positive aggregates. Following the first in-human study, results from the 11 patients who were enrolled in an investigator-initiated treatment program have provided evidence of the safety and therapeutic potential of jacifusen [146]. Serial intrathecal administration of the drug decreased *FUS* mRNA and protein levels and reduced CSF NfL concentration by up to 82.8%. One patient showed exceptional functional recovery in fine motor, gross motor, and respiratory domains. The results of the ongoing phase 1–3 clinical trial (Fusion, NCT04768972) will evaluate the clinical efficacy of jacifusen in FUS-ALS patients worldwide.

#### 4.2.3. C9orf72

Autosomal-dominantly inherited mutations in *C9orf72* were discovered later than other ALS related genes were and represent the most common genetic cause of ALS [147,148]. Hexanucleotide repeat (GGGGCC) expansion in the first intron or the promoter region of *C9orf72* is responsible for 40% of fALS and 7% of sALS cases [147,149,150]. Healthy individuals carry up to 8 repeats, and 24–29 repeats are considered as a risk factor. Thirty repeats are considered a pathogenic threshold, and patients can have up to thousands of repeats. [151,152,153]. C9orf72 pathology is suggested to be caused by both the loss and gain of protein function. Loss of function may occur due to haploinsufficiency resulting from decreased *C9orf72* gene expression and disrupted vesicular trafficking [154,155]. Gain-of-function toxicity may occur by two mechanisms: 1. mutant mRNA generation of nuclear foci and sequestration of essential RNA-binding proteins, which may impair their function [156]; 2. when sense and antisense strands of *C9orf72* RNA are translated by repeat-associated non-ATG translation and generate toxic aggregates of dipeptide repeat proteins (sense: polyGR, polyGA, polyGP; antisense: polyGP, polyPR, polyPA) [157,158].

Gene targeted therapy strategies to treat C9orf72-ALS include a targeted reduction in hexanucleotide repeat-containing *C9orf72* transcripts and dipeptide repeat proteins. The gene has three transcript variants called V1 (short protein isoform), V2 and V3 (full-length protein) [159]. Hexanucleotide repeats are located between non-coding exons 1a and 1b, and exon two has the translational start site (ATG). V1 and V3 transcripts have exons starting from exon 1a and the hexanucleotide repeat sequence, but the V2 transcript includes exons starting from exon 1b, which resides downstream of the hexanucleotide repeat. One therapeutic strategy under investigation involves ASOs that selectively reduce the V1 and V3 transcripts without impacting transcript V2. A proof-of-concept study has shown that Afinersen (ASO 5-2) was able to degrade both V1 and V3 transcripts by RNase-H1-mediated degradation in C9orf72 disease models, including patient-derived cells, mice, sheep, and monkeys. Encouraging results have led to the treatment of a single patient. The intrathecally injected Afinersen was well tolerated, the ALSFRS-R score improved, and dipeptide repeat proteins were reduced by 80% in CSF [159]. A larger clinical trial is needed to evaluate the efficacy of Afinersen.

BIIB078 (IONIS-C9Rx/Tadnersen) is another investigational ASO that selectively targets exon 1a-initiated transcripts V1 and V3 without altering V2 transcript levels. When the ASO was administered to mice expressing *C9orf72* RNAs with up to 450 GGGGCC repeats or with one or both *C9orf72* alleles inactivated, it reduced RNA foci, poly-glycine-proline (polyGP) and poly-glycine-alanine (polyGA) dipeptide-repeat proteins and improved behavioral deficits [160]. In a phase I study (NCT04288856) of adult patients with C9orf72-associated ALS, although CSF polyGP and polyGA levels were decreased as a result of target engagement, a high dose of BIIB078 administration caused an increase in CSF and plasma NfL concentrations and it did not improve clinical outcome measures including ALSFRS-R, respiratory function (SVC), muscle strength (HHD), and tongue strength (IOPI) [161]. No treatment-limiting safety concerns were raised; however, the trial was terminated by the sponsor, and potential challenges have been identified, including the heterogeneity of pathobiology in C9orf72-ALS, the importance of incorporating biomarkers of target engagement, and the need for further understanding of pathogenic effects of antisense oligonucleotides. Ongoing analysis of postmortem tissues from trial participants are expected to provide further insight into disease pathophysiology and the effects of BIIB078. Collectively, these studies and trials will help lay the foundation for the development of effective treatments in the future.

#### 4.2.4. TARDBP

Another common gene mutated in ALS is *TARDBP*. Since its discovery in 2006, more than 50 mutations have been reported and are responsible for 3.3% of fALS (autosomal dominant inheritance) and 0.5% of sALS [162]. TAR DNA-binding protein 43 (TDP-43) is a ubiquitously expressed and tightly regulated DNA/RNA-binding protein encoded by the *TARDBP* gene. It has roles in several cellular mechanisms, including RNA biogenesis, processing, and translation [163,164]. TDP-43 is predominantly localized in the nucleus under physiological conditions; however, in approximately 97% of ALS cases—regardless of *TARDBP* mutation status—the protein mislocalizes to the cytoplasm and forms aggregates in the brain and spinal cord, representing a pathological hallmark of the disease [162,164,165,166]. TDP-43-related pathology is suggested to be caused by both loss of TDP-43 function in the nucleus and gain of function in the cytoplasm [167]. Different strategies have been developed to target pathological forms of TDP-43 and to restore its homeostasis [168]. A recent study in ALS/FTD mice showed the potential of a gapmer-type ASO against human TDP-43 as a possible disease-modifying therapy [169]. Given that TDP-43 pathology represents a hallmark of ALS, alternative strategies have also been developed to target shared disease mechanisms rather than gene-specific mutations [167]. These include both upstream and downstream modulators of TDP-43 pathology. Ataxin-2 is an upstream modifier, where an increase in CAG repeats (27–33 repeats) in the gene has been reported to increase ALS disease risk by 11-fold [161]. ASO (BIIB105/ION541) or AAV-based delivery of RNAi (miRNA) targeting ataxin-2 in TDP-43 mice has shown promising results. These strategies were able to decrease TDP-43 aggregation, improve motor function, and markedly extend survival [170,171]. A phase I/II study (NCT04494256) with the ataxin-2 targeting ASO drug BIIB105 was ongoing but recently terminated since treatment did not reduce plasma NfL or impact clinical outcomes [172].

Stathmin-2 (*STMN2*) is a downstream effector of TDP-43 pathology. STMN2 is a tubulin-binding protein that is required for axon outgrowth, maintenance, and regeneration [173,174,175]. TDP-43 regulates pre-mRNA processing and is required for functional full-length stathmin-2 protein synthesis [176]. In healthy individuals, TDP-43 sterically blocks a cryptic splice site that resides in the first intron of the *STMN2* gene. When nuclear TDP-43 is lost in ALS patients, the cryptic splice site usage results in the inclusion of exon2a, which introduces an early stop codon and premature polyadenylation signal. This results in truncated protein synthesis and stathmin-2 loss of function. The ASO drug QRL-201 is developed to target the regulatory elements that TDP-43 regulates in *STMN2* pre-mRNA processing, restore functional stathmin-2 protein, and rescue TDP-43-related axonal regeneration [172]. A phase I study of QRL-201 (NCT05633459) is ongoing [177].

## 5. Clinical Trial Readiness

The last decade has witnessed the potential of gene targeted therapies for several disorders and clinical trials are now designed in a personalized way. A recent study highlighted that gene targeted therapies possess a higher rate of clinical development success compared to other therapeutic modalities [178]. Despite advances in tools and technologies enabling gene targeted therapies, translating these innovations into effective treatments remains challenging. The biological complexity and genetic and phenotypic heterogeneity of neurodegenerative diseases limit our understanding of disease mechanisms and natural history and makes clinical trial design and evaluation difficult. Consequently, clinical trial readiness remains a key determinant of success. Overcoming these challenges requires a multifaceted approach encompassing the use of disease-relevant models, natural history characterization and the development of robust biomarkers and clinical outcome measures.

Disease models are important resources for studying disease pathophysiology, biomarker discovery and preclinical studies for therapy development. Better understanding of disease and early diagnosis is vital, as experience has shown the importance of early therapeutic intervention in SMA before significant motor neuron loss occurs [179]. While the monogenic nature of SMA allows for early diagnosis through newborn screening programs, ALS is a genetically heterogeneous and mostly sporadic disease with no validated biomarkers for presymptomatic detection. Therefore, using ALS models is important for studies to understand disease etiology and progression, identify biomarkers for presymptomatic diagnosis, and evaluating early therapeutic efficacy. Animal models have been used as the gold standard in preclinical trials, though they have limitations. These model systems may not fully recapitulate the disease phenotype due to the complexity of human neuroanatomy, and evaluation of safety could be difficult. Interspecies differences are also a concern for gene targeted therapies, as species-specific gene editing efficacy in mice compared to primates has been reported [180]. In this sense, humanized mouse models and large non-human primates are pharmacologically more relevant disease models for dosing, efficacy, and safety studies [181,182]. However, the cost, time requirement, and inefficiency of genetic manipulations of large animals limit their widespread use. Another limitation is the diverse genetic and ethnic backgrounds of patients.

In this context, patient-derived cell models have been used as a valuable resource to understand disease mechanisms with the advantage of having the genetic background of the patient. In diseases with high genetic heterogeneity, such as ALS, the specific genes and mutations causing disease can vary. As a result, generalizing findings from a single animal model may lead to oversight of certain gene and mutation-specific phenotypes. Fibroblast cells have been used for studying disease mechanisms since they share similar gene expression profiles with neuronal cells. However, they are not the primary cell types affected in neurological disorders, which may limit the observations in a cell type-specific manner [183]. Induced pluripotent stem cells (iPSCs) are invaluable resources as they have the potential to differentiate into disease-relevant neuronal and glial cell types [184,185]. Generation of isogenic cell lines by correcting the patient’s mutation would eliminate the genetic background differences and help to elucidate the molecular mechanisms caused by a specific mutation. The Answer ALS platform provides a large set of data from patient and control iPSC-derived motor neurons as well as resources for studying gender involvement, dysregulated genes, and disrupted pathways in disease pathology [117,186]. Combining gene editing technologies such as CRISPR with stem cell technologies including organoids and organs-on-a-chip allows for the generation of disease models that can provide new insights into disease mechanisms and allow for drug testing in a human-relevant context [187]. A recent study in an SMA spinal cord organoid (SCO) model was generated using patient-derived iPSCs and isogenic controls. This study reported early neurodevelopmental defects in the disease pathogenesis, which might support even earlier therapeutic intervention in patients [188]. Early treatment of the SCO model with an SMN-restoring ASO rescued morphological and functional deficits as well as SMN-dependent splicing defects [189]. Neuromuscular organoids, 3D human cortical organoid slice culture models, and presymptomatic cerebral organoids were derived from iPSCs of C9orf72-ALS patients and were able to recapitulate spinal neuromuscular pathologies, early changes in 3D human brain tissue organization, synaptic structure and function, and mature astroglial/neuronal phenotypes [190,191,192]. Although each model has its own limitations, these tools provide opportunities to deepen our understanding of disease mechanisms and enable the development of effective treatment for neurodegenerative disorders.

Besides the need for disease-relevant models, the natural history of disease is crucial for facilitating clinical trial readiness. The natural history of a disease is traditionally defined as the course a disease takes in the absence of intervention in individuals with the disease, from the disease’s onset until either the disease’s resolution or the individual’s death [193]. Natural history studies provide detailed information on initial disease symptoms, disease progression, and severity. This knowledge is valuable for identifying patient subpopulations within a heterogeneous cohort and can help recognize subgroups that may benefit from a specific treatment. In addition, natural history studies allow for the development of clinical outcome assessments for identifying disease features, monitoring and managing the clinical status of the disease, and testing drug efficacy in clinical trials [194,195]. Patient-reported outcome measurements are also important to identify the importance and prevalence of key symptoms and factors linked to disease severity [174]. Natural history studies allow for the collection of blood, tissue, or bodily fluid specimens and imaging (e.g., magnetic resonance imaging of brain and skeletal muscle), which will help in identifying and validating biomarkers for diagnostic, prognostic, or predictive determinations for drug treatment [195,196,197]. Once validated, these biomarkers can serve as endpoints to ensure target engagement in clinical trials, aid efficient trial design and accelerate the regulatory decision-making. Accordingly, in recent years, NfL and phosphorylated neurofilament heavy-chain (pNfH) proteins have been identified as biomarkers for neuron degeneration and drug response for several neurodegenerative disorders. NfL has been accepted as a surrogate endpoint and played a key role in the approval of Tofersen for SOD1-ALS. Broadening the biomarker repertoire—through cross-disease validation of existing biomarkers and inclusion of new ones—alongside integration of molecular and non-molecular measures (e.g., electrophysiological or imaging) can significantly enhance clinical trial design and interpretation [198,199]. Finally, it is essential to emphasize the critical role of patient registries in establishing outcome measures and endpoints, in addition to supporting patient recruitment for clinical trials. Together, while not limited to these components, the coordinated use of predictive disease models, well-characterized natural history data, and validated biomarkers will be central to achieving more efficient and lower-risk clinical trial design in neurodegenerative disorders.

## 6. Future Directions

Looking forward, advancements in precision medicine and emerging technologies will offer promising gene targeted therapy opportunities for various disorders. Genetic screening will play a critical role in addressing the genetic heterogeneity of diseases such as ALS, enabling the development of tailored therapies for specific mutations [115]. Gene editing technologies, particularly CRISPR-based approaches, will remain integral in the development of disease models and precise therapies for various disorders. Casgevy (NCT03745287) and Lyfgenia (NTC04293185) are the first examples of approved CRISPR-Cas9 gene editing therapies for sickle cell disease and beta thalassemia, showing the substantial potential of these approaches. Gene editing approaches are already being explored in SMA and ALS, demonstrating the potential of these therapies to offer effective treatments. Adenosine base editors have demonstrated the potential to correct C to T transition in *SMN2*, allowing exon 7 retention and increasing SMN levels in patient-derived fibroblasts and mice, with no off-target effects. This correction resulted in improved motor function and extended survival in animal models [200,201]. Base editing of *SMN2* allows for the endogenous regulation of the SMN protein, which could eliminate potential toxicity due to the constitutive expression driven by onasemnogene abeparvovec-xioi (Zolgensma). RNA editing is also receiving attention due to safety concerns associated with editing DNA. Recent studies suggest that this approach should be considered for ALS. CRISPR-Cas13d variants have been shown to significantly reduce *C9orf72* sense and antisense repeat transcripts in patient-derived iPSC-neuron lines and different *C9orf72* mouse models without affecting V2 transcript levels [202,203]. As the technology continues to evolve, integration of artificial intelligence and machine learning will expect to improve CRISPR systems by optimizing guide RNA design, selecting optimal Cas variants, and reducing off-target effects [204].

Future therapies in ALS would also benefit from convergent pathological pathways between sALS and fALS to allow for the targeting of a broader range of patients [205]. Key pathways—including glutamate excitotoxicity, neuronal hyperexcitability, oxidative stress, mitochondrial dysfunction, protein misfolding, impaired autophagy, trophic factor signaling, and inflammatory responses—represent promising targets that could be revisited and explored in combinatorial treatment approaches [206]. Skeletal muscle has also emerged as a relevant therapeutic target in both ALS and SMA. In SMA, disease-modifying therapies have significantly altered the natural history of the disease; however, some patients continue to exhibit muscle weakness, highlighting the need for long-term functional support [207]. Consequently, patients may benefit from combinatorial approaches that couple SMN-restoring therapies with muscle-targeted interventions. Improved experimental models that recapitulate neuromuscular interactions will be essential to better define the individual and combined contributions of neurons and muscle to disease pathology and identify novel therapeutic targets [206,207].

Future progress in these strategies will critically depend on overcoming the current limitations like optimizing delivery routes and systems, precise dosing, immune responses and toxicity, and developing faster, less invasive and cost-effective approaches. One of the major challenges in motor neuron disorders remains the efficient delivery of therapeutic molecules to the CNS, which can be achieved through either systemic or direct CNS-targeted approaches. Intravenous administration represents a systemic delivery method that is less invasive; however, its CNS biodistribution is restricted by BBB, which requires the administration of higher doses, increasing the risk of toxicity. In contrast, direct CNS delivery methods—including intrathecal (IT), intracerebroventricular (ICV), intraparenchymal, subpial, and nose-to-brain routes—bypass the BBB and enable localized delivery [208]. These approaches achieve higher local concentrations with lower doses, reducing systemic toxicity and off-target effects while improving distribution to the target cells. However, direct CNS delivery is more invasive and may involve procedural risks. Among these, intrathecal delivery has emerged as the most used route due to its relatively lower invasiveness and feasibility in outpatient settings, as demonstrated by therapies such as Nusinersen and Tofersen. Despite these advances, significant challenges remain, particularly in achieving cell-specific targeting and developing less invasive delivery strategies. Furthermore, the complexity of neurological disorders necessitates a deeper understanding of disease-specific factors, as the physiological environment—including cerebrospinal fluid dynamics, BBB integrity, and cellular uptake mechanisms—can vary significantly depending on the pathological state [208].

While several gene targeted therapies have demonstrated clinical benefit, it remains challenging based on current knowledge, to prioritize one approach over another in terms of clinical efficacy and safety. Each strategy represents distinct advantages and limitations, and the complexity and heterogeneity of motor neuron disorders further preclude the clear superiority of any single modality. Emerging approaches may hold promise; however, improvements will depend on overcoming current limitations, deepening our understanding of disease pathology and the mechanisms underlying therapeutic responses, and strengthening the evaluation of target engagement and therapeutic efficacy using robust, standardized biomarkers, biochemical, and imaging-based measures. Taken together, advances in gene editing and combinatorial therapeutic strategies along with efforts to address key biological, technical, and translational bottlenecks hold significant promise for improving the precision, effectiveness, and durability of therapies for neurodegenerative diseases.

## Figures and Tables

**Table 1:** Current ALS gene targeted therapies in clinical trials.

Interventions	Target	Modality	National Clinical Trial Identifier	Study Status
BIIB067 (Tofersen)	*SOD1*	ASO	NCT04856982	Phase III—Active, not recruiting. Approved for sale to the public (Expanded Access Program-NCT04972487)
			NCT03070119	Phase III—Completed
			NCT02623699	Phase III—Completed
			NCT03764488	Phase I—Completed
RAG-17	*SOD1*	RNAi	NCT05903690	Early Phase I—completed
AMT-162 (APB-102)	*SOD1*	AAV-RNAi (miRNA)	NCT06100276	Phase I/II—Active, not recruiting
ION363 (Jacifusen)	*FUS*	ASO	NCT04768972	Phase III—Active, not recruiting
Engensis (VM202)	*HGF* addition	Non-viral plasmid gene delivery	NCT04632225	Phase IIa—Completed
			NCT05176093	Phase IIa—Completed
			NCT02039401	Phase I/II—Completed
QRL-201	*STMN2*	ASO	NCT05633459	Phase I—Active, not recruiting

Abbreviations: ASO, antisense oligonucleotide; RNAi, RNA interference; *HGF*, Hepatocyte growth factor; *STMN2*, Stathmin 2; *FUS*, Fused in sarcoma; *SOD1*, Superoxide dismutase 1. Sourced from [119].

## Data Availability

No new data were created or analyzed in this study.

## Abbreviations

The following abbreviations are used in this manuscript:

AAV	Adeno-associated virus
AAV9	Adeno-associated virus serotype 9
Ad	Adenovirus
ALS	Amyotrophic lateral sclerosis
ALSFRS-R	Amyotrophic Lateral Sclerosis Functional Rating Scale–Revised
ASO	Antisense oligonucleotide
C9orf72	Chromosome 9 open reading frame 72
CAR-T	Chimeric antigen receptor T-cell
Cas9	CRISPR-associated protein 9
Cas13d	CRISPR-associated protein 13d
CNS	Central nervous system
CRISPR	Clustered Regularly Interspaced Short Palindromic Repeats
CSF	Cerebrospinal fluid
DNA	Deoxyribonucleic acid
dsDNA	Double-stranded DNA
EMA	European Medicines Agency
fALS	Familial amyotrophic lateral sclerosis
FDA	Food and Drug Administration
FTD	Frontotemporal dementia
FUS	Fused in sarcoma
gRNA	Guide RNA
HR	Homologous recombination
iPSC	Induced pluripotent stem cell
ITR	Inverted terminal repeat
LTR	Long terminal repeat
miRNA	MicroRNA
mRNA	Messenger ribonucleic acid
NDDs	Neurodegenerative diseases
NfL	Neurofilament light chain
NHEJ	Non-homologous end joining
RNA	Ribonucleic acid
RNAi	RNA interference
RNaseH1	Ribonuclease H1
sALS	Sporadic amyotrophic lateral sclerosis
scAAV	Self-complementary adeno-associated virus
sgRNA	Single guide RNA
siRNA	Small interfering RNA
SMA	Spinal muscular atrophy
SMN	Survival motor neuron
SMN1	Survival motor neuron 1
SMN2	Survival motor neuron 2
SOD1	Superoxide dismutase 1
STMN2	Stathmin-2
TALEN	Transcription activator-like effector nuclease
TARDBP	TAR DNA-binding protein
TDP-43	TAR DNA-binding protein 43
UTR	Untranslated region
ZFNs	Zinc finger nucleases

## References

[1] Arabi F., Mansouri V., Ahmadbeigi N. (2022). Gene therapy clinical trials, where do we go? An overview. Biomed. Pharmacother..

[2] U.S. Food and Drug Administration (FDA) What Is Gene Therapy?. https://www.fda.gov/vaccines-blood-biologics/cellular-gene-therapy-products/what-gene-therapy.

[3] World Health Organization (WHO) International Clinical Trials Registry Platform (ICTRP). https://trialsearch.who.int/Default.aspx.

[4] Ma C.C., Wang Z.L., Xu T., He Z.Y., Wei Y.Q. (2020). The approved gene therapy drugs worldwide: From 1998 to 2019. Biotechnol. Adv..

[5] U.S. Food and Drug Administration (FDA) Purple Book Database. https://purplebooksearch.fda.gov/.

[6] European Medicines Agency (EMA). https://www.ema.europa.eu/en/medicines.

[7] Razi Soofiyani S., Baradaran B., Lotfipour F., Kazemi T., Mohammadnejad L. (2013). Gene therapy, early promises, subsequent problems, and recent breakthroughs. Adv. Pharm. Bull..

[8] Seow Y., Wood M.J. (2009). Biological gene delivery vehicles: Beyond viral vectors. Mol. Ther..

[9] Alhakamy N.A., Curiel D.T., Berkland C.J. (2021). The era of gene therapy: From preclinical development to clinical application. Drug Discov. Today.

[10] Shin M.H., He Y., Marrogi E., Piperdi S., Ren L., Khanna C., Gorlick R., Liu C., Huang J. (2016). A RUNX2-mediated epigenetic regulation of the survival of p53 defective cancer cells. PLoS Genet..

[11] Zuris J.A., Thompson D.B., Shu Y., Guilinger J.P., Bessen J.L., Hu J.H., Maeder M.L., Joung J.K., Chen Z.-Y., Liu D.R. (2015). Cationic lipid-mediated delivery of proteins enables efficient protein-based genome editing in vitro and in vivo. Nat. Biotechnol..

[12] Wang M., Zuris J.A., Meng F., Rees H., Sun S., Deng P., Han Y., Gao X., Pouli D., Wu Q. (2016). Efficient delivery of genome-editing proteins using bioreducible lipid nanoparticles. Proc. Natl. Acad. Sci. USA.

[13] Li C., Samulski R.J. (2020). Engineering adeno-associated virus vectors for gene therapy. Nat. Rev. Genet..

[14] Bulcha J.T., Wang Y., Ma H., Tai P.W.L., Gao G. (2021). Viral vector platforms within the gene therapy landscape. Signal Transduct. Target. Ther..

[15] Alba R., Bosch A., Chillon M. (2005). Gutless adenovirus: Last-generation adenovirus for gene therapy. Gene Ther..

[16] Atchison R.W., Casto B.C., Hammon W.M. (1965). Adenovirus-associated defective virus particles. Science.

[17] Wang J.H., Gessler D.J., Zhan W., Gallagher T.L., Gao G. (2024). Adeno-associated virus as a delivery vector for gene therapy of human diseases. Signal Transduct. Target. Ther..

[18] Penaud-Budloo M., Le Guiner C., Nowrouzi A., Toromanoff A., Chérel Y., Chenuaud P., Schmidt M., von Kalle C., Rolling F., Moullier P. (2008). Adeno-associated virus vector genomes persist as episomal chromatin in primate muscle. J. Virol..

[19] Ling Q., Herstine J.A., Bradbury A., Gray S.J. (2023). AAV-based in vivo gene therapy for neurological disorders. Nat. Rev. Drug Discov..

[20] Pupo A., Fernández A., Low S.H., François A., Suárez-Amarán L., Samulski R.J. (2022). AAV vectors: The Rubik’s cube of human gene therapy. Mol. Ther..

[21] Suarez-Amaran L., Song L., Tretiakova A.P., Mikhail S.A., Samulski R.J. (2025). AAV vector development, back to the future. Mol. Ther..

[22] Riyad J.M., Weber T. (2021). Intracellular trafficking of adeno-associated virus (AAV) vectors: Challenges and future directions. Gene Ther..

[23] van Haasteren J., Li J., Scheideler O.J., Murthy N., Schaffer D.V. (2020). The delivery challenge: Fulfilling the promise of therapeutic genome editing. Nat. Biotechnol..

[24] Cichon M., Jozkowicz A., Grochot-Przeczek A. (2026). Advances in Endothelial Cell Targeting by AAV Vectors. Acta Biochim. Pol..

[25] Ramamoorth M., Narvekar A. (2015). Non viral vectors in gene therapy—An overview. J. Clin. Diagn. Res..

[26] Picanco-Castro V., Pereira C.G., Covas D.T., Porto G.S., Athanassiadou A., Figueiredo M.L. (2020). Emerging patent landscape for non-viral vectors used for gene therapy. Nat. Biotechnol..

[27] Shahryari A., Burtscher I., Nazari Z., Lickert H. (2021). Engineering gene therapy: Advances and barriers. Adv. Ther..

[28] Zhou H., He Y., Xiong W., Jing S., Duan X., Huang Z., Nahal G.S., Peng Y., Li M., Zhu Y. (2023). MSC based gene delivery methods and strategies improve the therapeutic efficacy of neurological diseases. Bioact. Mater..

[29] Zu H., Gao D. (2021). Non-viral vectors in gene therapy: Recent development, challenges, and prospects. AAPS J..

[30] Devinsky O., Coller J., Ahrens-Nicklas R., Liu X.S., Ahituv N., Davidson B.L., Bishop K.M., Weiss Y., Mingorance A. (2025). Gene Therapies for Neurogenetic Disorders. Trends Mol. Med..

[31] Giovannelli I., Higginbottom A., Kirby J., Azzouz M., Shaw P.J. (2023). Prospects for gene replacement therapies in amyotrophic lateral sclerosis. Nat. Rev. Neurol..

[32] Goswami R., Subramanian G., Silayeva L., Newkirk I., Doctor D., Chawla K., Chattopadhyay S., Chandra D., Chilukuri N., Betapudi V. (2019). Gene therapy leaves a vicious cycle. Front. Oncol..

[33] Poletto E., Baldo G., Gomez-Ospina N. (2020). Genome editing for mucopolysaccharidoses. Int. J. Mol. Sci..

[34] Sun J., Roy S. (2021). Gene-based therapies for neurodegenerative diseases. Nat. Neurosci..

[35] Kim H., Kim J.S. (2014). A guide to genome engineering with programmable nucleases. Nat. Rev. Genet..

[36] Jinek M., Chylinski K., Fonfara I., Hauer M., Doudna J.A., Charpentier E. (2012). A programmable dual-RNA-guided DNA endonuclease in adaptive bacterial immunity. Science.

[37] Mojica F.J., Díez-Villaseñor C., García-Martínez J., Soria E. (2005). Intervening sequences of regularly spaced prokaryotic repeats derive from foreign genetic elements. J. Mol. Evol..

[38] Barrangou R., Fremaux C., Deveau H., Richards M., Boyaval P., Moineau S., Romero D.A., Horvath P. (2007). CRISPR provides acquired resistance against viruses in prokaryotes. Science.

[39] Wang J.Y., Doudna J.A. (2023). CRISPR technology: A decade of genome editing is only the beginning. Science.

[40] Jiang F., Doudna J.A. (2017). CRISPR–Cas9 structures and mechanisms. Annu. Rev. Biophys..

[41] Casas-Mollano J.A., Zinselmeier M.H., Erickson S.E., Smanski M.J. (2020). CRISPR-Cas activators for engineering gene expression in higher eukaryotes. CRISPR J..

[42] Qi L.S., Larson M.H., Gilbert L.A., Doudna J.A., Weissman J.S., Arkin A.P., Lim W.A. (2013). Repurposing CRISPR as an RNA-guided platform for sequence-specific control of gene expression. Cell.

[43] Komor A.C., Kim Y.B., Packer M.S., Zuris J.A., Liu D.R. (2016). Programmable editing of a target base in genomic DNA without double-stranded DNA cleavage. Nature.

[44] Kang Y., Chu C., Wang F., Niu Y. (2019). CRISPR/Cas9-mediated genome editing in nonhuman primates. Dis. Model Mech..

[45] Chen P.J., Liu D.R. (2023). Prime editing for precise and highly versatile genome manipulation. Nat. Rev. Genet..

[46] Newby G.A., Liu D.R. (2021). In vivo somatic cell base editing and prime editing. Mol. Ther..

[47] Anzalone A.V., Randolph P.B., Davis J.R., Sousa A.A., Koblan L.W., Levy J.M., Chen P.J., Wilson C., Newby G.A., Raguram A. (2019). Search-and-replace genome editing without double-strand breaks or donor DNA. Nature.

[48] Chauhan V.P., Sharp P.A., Langer R. (2025). Engineered prime editors with minimal genomic errors. Nature.

[49] Lee J., Kweon J., Kim Y. (2025). Emerging trends in prime editing for precision genome editing. Exp. Mol. Med..

[50] Yashooa R.K., Nabi A.Q., Smail S.W., Azeez S.S., Nooh W.A., Mustafa S.A., Al-Farha A.A.-B., Capitanio N., Shekha M.S. (2026). CRISPR–Cas technologies in neurodegenerative disorders: Mechanistic insights, therapeutic potential, and translational challenges. Front. Neurol..

[51] Zhao Z., Shang P., Mohanraju P., Geijsen N. (2023). Prime editing: Advances and therapeutic applications. Trends Biotechnol..

[52] Schoch K.M., Miller T.M. (2017). Antisense oligonucleotides: Translation from mouse models to human neurodegenerative diseases. Neuron.

[53] Dudzisz K., Wandzik I. (2025). Antisense oligonucleotides: A promising advancement in neurodegenerative disease treatment. Eur. J. Pharmacol..

[54] Bennett C.F., Kordasiewicz H.B., Cleveland D.W. (2021). Antisense drugs make sense for neurological diseases. Annu. Rev. Pharmacol. Toxicol..

[55] Marrosu E., Ala P., Muntoni F., Zhou H. (2017). Gapmer antisense oligonucleotides suppress the mutant allele of COL6A3 and restore functional protein in Ullrich muscular dystrophy. Mol. Ther. Nucleic Acids.

[56] Wu H., Lima W.F., Zhang H., Fan A., Sun H., Crooke S.T. (2004). Determination of the role of the human RNase H1 in the pharmacology of DNA-like antisense drugs. J. Biol. Chem..

[57] Lima W.F., De Hoyos C.L., Liang X.H., Crooke S.T. (2016). RNA cleavage products generated by antisense oligonucleotides and siRNAs are processed by the RNA surveillance machinery. Nucleic Acids Res..

[58] Lauffer M.C., van Roon-Mom W., Aartsma-Rus A., Collaborative N. (2024). Possibilities and limitations of antisense oligonucleotide therapies for the treatment of monogenic disorders. Commun. Med..

[59] Amado D.A., Davidson B.L. (2021). Gene therapy for ALS: A review. Mol. Ther..

[60] Brillante S., Volpe M., Indrieri A. (2024). Advances in microRNA therapeutics: From preclinical to clinical studies. Hum. Gene Ther..

[61] Borel F., Kay M.A., Mueller C. (2014). Recombinant AAV as a platform for translating the therapeutic potential of RNA interference. Mol. Ther..

[62] Sumner C.J., Miller T.M. (2024). The expanding application of antisense oligonucleotides to neurodegenerative diseases. J. Clin. Investig..

[63] Bhati V., Prasad S., Kabra A. (2025). RNA-Based Therapies for Neurodegenerative Disease: Targeting Molecular Mechanisms for Disease Modification. Mol. Cell. Neurosci..

[64] Bennett C.F. (2019). Therapeutic antisense oligonucleotides are coming of age. Annu. Rev. Med..

[65] Leung A.K., Tam Y.Y., Cullis P.R. (2014). Lipid nanoparticles for short interfering RNA delivery. Adv. Genet..

[66] Dahlman J.E., Kauffman K.J., Langer R., Anderson D.G. (2014). Nanotechnology for in vivo targeted siRNA delivery. Adv. Genet..

[67] Geary R.S., Norris D., Yu R., Bennett C.F. (2015). Pharmacokinetics, biodistribution and cell uptake of antisense oligonucleotides. Adv. Drug Deliv. Rev..

[68] Garreau M., Weidner J., Hamilton R., Kolosionek E., Toki N., Stavenhagen K., Paris C., Bonetti A., Czechtizky W., Gnerlich F. (2024). Chemical modification patterns for microRNA therapeutic mimics: A structure-activity relationship (SAR) case-study on miR-200c. Nucleic Acids Res..

[69] Wang P., Zhou Y., Richards A.M. (2021). Effective tools for RNA-derived therapeutics: siRNA interference or miRNA mimicry. Theranostics.

[70] Alterman J.F., Godinho B.M.D.C., Hassler M.R., Ferguson C.M., Echeverria D., Sapp E., Haraszti R.A., Coles A.H., Conroy F., Miller R. (2019). A divalent siRNA chemical scaffold for potent and sustained modulation of gene expression throughout the central nervous system. Nat. Biotechnol..

[71] Zhang X. (2024). Splice-Switching Antisense Oligonucleotides for Pediatric Neurological Disorders. Front. Mol. Neurosci..

[72] McDowall S., Aung-Htut M., Wilton S., Li D. (2024). Antisense Oligonucleotides and Their Applications in Rare Neurological Diseases. Front. Neurosci..

[73] Chen S., Heendeniya S.N., Le B.T., Rahimizadeh K., Rabiee N., Zahra Q.U.A., Veedu R.N. (2024). Splice-Modulating Antisense Oligonucleotides as Therapeutics for Inherited Metabolic Diseases. BioDrugs.

[74] Rinaldi C., Wood M.J.A. (2018). Antisense oligonucleotides: The next frontier for treatment of neurological disorders. Nat. Rev. Neurol..

[75] Havens M.A., Hastings M.L. (2016). Splice-switching antisense oligonucleotides as therapeutic drugs. Nucleic Acids Res..

[76] Wirth B. (2021). Spinal muscular atrophy: In the challenge lies a solution. Trends Neurosci..

[77] Yeo C.J.J., Tizzano E.F., Darras B.T. (2024). Challenges and opportunities in spinal muscular atrophy therapeutics. Lancet Neurol..

[78] Keinath M.C., Prior D.E., Prior T.W. (2021). Spinal muscular atrophy: Mutations, testing, and clinical relevance. Appl. Clin. Genet..

[79] Riessland M., Kaczmarek A., Schneider S., Swoboda K.J., Löhr H., Bradler C., Grysko V., Dimitriadi M., Hosseinibarkooie S., Torres-Benito L. (2017). Neurocalcin delta suppression protects against spinal muscular atrophy in humans and across species by restoring impaired endocytosis. Am. J. Hum. Genet..

[80] Oprea G.E., Kröber S., McWhorter M.L., Rossoll W., Müller S., Krawczak M., Bassell G.J., Beattie C.E., Wirth B. (2008). Plastin 3 is a protective modifier of autosomal recessive spinal muscular atrophy. Science.

[81] Shababi M., Habibi J., Yang H.T., Vale S.M., Sewell W.A., Lorson C.L. (2010). Cardiac defects contribute to the pathology of spinal muscular atrophy models. Hum. Mol. Genet..

[82] Allardyce H., Kuhn D., Hernandez-Gerez E., Hensel N., Huang Y.-T., Faller K., Gillingwater T.H., Quondamatteo F., Claus P., Parson S.H. (2020). Renal pathology in a mouse model of severe spinal muscular atrophy is associated with downregulation of glial cell-line derived neurotrophic factor (GDNF). Hum. Mol. Genet..

[83] Somers E., Lees R.D., Hoban K., Sleigh J.N., Zhou H., Muntoni F., Talbot K., Gillingwater T.H., Parson S.H. (2016). Vascular defects and spinal cord hypoxia in spinal muscular atrophy. Ann. Neurol..

[84] Hensel N., Claus P. (2018). The actin cytoskeleton in SMA and ALS: How does it contribute to motoneuron degeneration?. Neuroscientist.

[85] Kaymaz A.Y., Bal S.K., Bora G., Talim B., Ozon A., Alikasifoglu A., Topaloglu H., Yurter H.E. (2022). Alterations in insulin-like growth factor system in spinal muscular atrophy. Muscle Nerve.

[86] Bora G., Hensel N., Rademacher S., Koyunoğlu D., Sunguroğlu M., Aksu-Mengeş E., Balcı-Hayta B., Claus P., Erdem-Yurter H. (2021). Microtubule-associated protein 1B dysregulates microtubule dynamics and neuronal mitochondrial transport in spinal muscular atrophy. Hum. Mol. Genet..

[87] Chaytow H., Huang Y.T., Gillingwater T.H., Faller K.M.E. (2018). The role of survival motor neuron protein (SMN) in protein homeostasis. Cell. Mol. Life Sci..

[88] Tapken I., Schweitzer T., Paganin M., Schüning T., Detering N.T., Sharma G., Niesert M., Saffari A., Kuhn D., Glynn A. (2025). The systemic complexity of a monogenic disease: The molecular network of spinal muscular atrophy. Brain.

[89] Özer P.Z., Koyunoğlu D., Son Ç.D., Erdem-Yurter H., Bora G. (2022). SMN loss dysregulates microtubule-associated proteins in spinal muscular atrophy model. Mol. Cell. Neurosci..

[90] Dayangaç-Erden D., Bora G., Ayhan P., Kocaefe Ç., Dalkara S., Yelekçi K., Demir A.S., Erdem-Yurter H. (2009). Histone deacetylase inhibition activity and molecular docking of (E)-resveratrol: Its therapeutic potential in spinal muscular atrophy. Chem. Biol. Drug Des..

[91] Chaytow H., Faller K.M.E., Huang Y.T., Gillingwater T.H. (2021). Spinal muscular atrophy: From approved therapies to future therapeutic targets for personalized medicine. Cell Rep. Med..

[92] Mercuri E., Pera M.C., Scoto M., Finkel R., Muntoni F. (2020). Spinal muscular atrophy—Insights and challenges in the treatment era. Nat. Rev. Neurol..

[93] Jablonka S., Hennlein L., Sendtner M. (2022). Therapy development for spinal muscular atrophy: Perspectives for muscular dystrophies and neurodegenerative disorders. Neurol. Res. Pract..

[94] Singh N.K., Singh N.N., Androphy E.J., Singh R.N. (2006). Splicing of a critical exon of human survival motor neuron is regulated by a unique silencer element located in the last intron. Mol. Cell. Biol..

[95] Hua Y., Vickers T.A., Okunola H.L., Bennett C.F., Krainer A.R. (2008). Antisense masking of an hnRNP A1/A2 intronic splicing silencer corrects SMN2 splicing in transgenic mice. Am. J. Hum. Genet..

[96] Biogen SPINRAZA^®^ (Nusinersen). https://www.spinraza.com/.

[97] Novartis Gene Therapies. ZOLGENSMA^®^ (Onasemnogene Abeparvovec-Xioi). https://www.zolgensma.com/.

[98] Thomsen G., Burghes A.H.M., Hsieh C., Do J., Chu B.T.T., Perry S., Barkho B., Kaufmann P., Sproule D.M., Feltner D.E. (2021). Biodistribution of onasemnogene abeparvovec DNA, mRNA and SMN protein in human tissue. Nat. Med..

[99] Roche FDA Approves Roche’s Evrysdi Tablet as First and only Tablet for Spinal Muscular Atrophy (SMA). https://www.roche.com/media/releases/med-cor-2025-02-12.

[100] Ottesen E.W., Singh N.N., Luo D., Kaas B., Gillette B.J., Seo J., Jorgensen H.J., Singh R.N. (2023). Diverse targets of SMN2-directed splicing-modulating small molecule therapeutics for spinal muscular atrophy. Nucleic Acids Res..

[101] Wirth B., Karakaya M., Kye M.J., Mendoza-Ferreira N. (2020). Twenty-five years of spinal muscular atrophy research: From phenotype to genotype to therapy, and what comes next. Annu. Rev. Genom. Hum. Genet..

[102] Singh R.N., Ottesen E.W., Singh N.N. (2020). The first orally deliverable small molecule for the treatment of spinal muscular atrophy. Neurosci. Insights.

[103] Chiriboga C.A., Bruno C., Duong T., Fischer D., Mercuri E., Kirschner J., Kostera-Pruszczyk A., Jaber B., Gorni K., Kletzl H. (2024). JEWELFISH: 24-month results from an open-label study in non-treatment-naïve patients with SMA receiving treatment with risdiplam. J. Neurol..

[104] Bekircan-Kurt C.E., Subramanian S., Chagat S., Mackenzie S.J., Iammarino M., Reash N., Richardson C., Tsao C., Noritz G., Gushue C. (2025). Transitioning from nusinersen to risdiplam for spinal muscular atrophy in clinical practice: A single-center experience. Muscle Nerve.

[105] Oechsel K.F., Cartwright M.S. (2021). Combination therapy with onasemnogene and risdiplam in spinal muscular atrophy type 1. Muscle Nerve.

[106] Cure SMA SMA Drug Pipeline. https://www.curesma.org/sma-drug-pipeline/.

[107] Belančić A., Eustaquio P., Gkrinia E.M.M., Stević I., Javor E., Lam Y.W., Janković S., Vitezić D. (2026). The evolving therapeutic landscape of spinal muscular atrophy—A scoping review of investigational agents, emerging delivery technologies and strategic innovations. Br. J. Clin. Pharmacol..

[108] Hardiman O., Al-Chalabi A., Chio A., Corr E.M., Logroscino G., Robberecht W., Shaw P.J., Simmons Z., van den Berg L.H. (2017). Amyotrophic lateral sclerosis. Nat. Rev. Dis. Primers.

[109] Van Damme P., Robberecht W., Van Den Bosch L. (2017). Modelling amyotrophic lateral sclerosis: Progress and possibilities. Dis. Model Mech..

[110] Shatunov A., Al-Chalabi A. (2021). The genetic architecture of ALS. Neurobiol. Dis..

[111] Chiò A., Logroscino G., Traynor B., Collins J., Simeone J., Goldstein L., White L. (2013). Global epidemiology of amyotrophic lateral sclerosis: A systematic review of the published literature. Neuroepidemiology.

[112] Xu L., Liu T., Liu L., Yao X., Chen L., Fan D., Zhan S., Wang S. (2020). Global variation in prevalence and incidence of amyotrophic lateral sclerosis: A systematic review and meta-analysis. J. Neurol..

[113] Logroscino G., Traynor B.J., Hardiman O., Chiò A., Mitchell D., Swingler R.J., Millul A., Benn E., Beghi E., Eurals F. (2010). Incidence of amyotrophic lateral sclerosis in Europe. J. Neurol. Neurosurg. Psychiatry.

[114] Ruffo P., Traynor B.J., Conforti F.L. (2025). Advancements in genetic research and RNA therapy strategies for amyotrophic lateral sclerosis (ALS): Current progress and future prospects. J. Neurol..

[115] Feldman E.L., Goutman S.A., Petri S., Mazzini L., Savelieff M.G., Shaw P.J., Sobue G. (2022). Amyotrophic lateral sclerosis. Lancet.

[116] Chia R., Chiò A., Traynor B.J. (2018). Novel genes associated with amyotrophic lateral sclerosis: Diagnostic and clinical implications. Lancet Neurol..

[117] Akçimen F., Lopez E.R., Landers J.E., Nath A., Chiò A., Chia R., Traynor B.J. (2023). Amyotrophic lateral sclerosis: Translating genetic discoveries into therapies. Nat. Rev. Genet..

[118] Renton A.E., Chiò A., Traynor B.J. (2014). State of play in amyotrophic lateral sclerosis genetics. Nat. Neurosci..

[119] ClinicalTrials.gov. https://clinicaltrials.gov/.

[120] Müller K., Brenner D., Weydt P., Grehl T., Petri S., Grosskreutz J., Schuster J., Volk A.E., Borck G., Kubisch C. (2018). Comprehensive analysis of the mutation spectrum in 301 German ALS families. J. Neurol. Neurosurg. Psychiatry.

[121] Sherman L., Dafni N., Lieman-Hurwitz J., Groner Y. (1983). Nucleotide sequence and expression of human chromosome 21-encoded superoxide dismutase mRNA. Proc. Natl. Acad. Sci. USA.

[122] Niwa J.-I., Yamada S.-I., Ishigaki S., Sone J., Takahashi M., Katsuno M., Tanaka F., Doyu M., Sobue G. (2007). Disulfide bond mediates aggregation, toxicity, and ubiquitylation of familial amyotrophic lateral sclerosis-linked mutant SOD1. J. Biol. Chem..

[123] Fridovich I. (1986). Biological effects of the superoxide radical. Arch. Biochem. Biophys..

[124] Saccon R.A., Bunton-Stasyshyn R.K., Fisher E.M., Fratta P. (2013). Is SOD1 loss of function involved in amyotrophic lateral sclerosis?. Brain.

[125] Lim C.K., Gapinske M., Brooks A.K., Woods W.S., Powell J.E., Zeballos C.M.A., Winter J., Perez-Pinera P., Gaj T. (2020). Treatment of a mouse model of ALS by in vivo base editing. Mol. Ther..

[126] Duan W., Guo M., Yi L., Liu Y., Li Z., Ma Y., Zhang G., Liu Y., Bu H., Song X. (2020). The deletion of mutant SOD1 via CRISPR/Cas9/sgRNA prolongs survival in an amyotrophic lateral sclerosis mouse model. Gene Ther..

[127] Smith R.A., Miller T.M., Yamanaka K., Monia B.P., Condon T.P., Hung G., Lobsiger C.S., Ward C.M., McAlonis-Downes M., Wei H. (2006). Antisense oligonucleotide therapy for neurodegenerative disease. J. Clin. Investig..

[128] Miller T.M., Pestronk A., David W., Rothstein J., Simpson E., Appel S.H., Andres P.L., Mahoney K., Allred P., Alexander K. (2013). An antisense oligonucleotide against SOD1 delivered intrathecally for patients with SOD1 familial amyotrophic lateral sclerosis: A phase 1, randomised, first-in-man study. Lancet Neurol..

[129] McCampbell A., Cole T., Wegener A.J., Tomassy G.S., Setnicka A., Farley B.J., Schoch K.M., Hoye M.L., Shabsovich M., Sun L. (2018). Antisense oligonucleotides extend survival and reverse decrement in muscle response in ALS models. J. Clin. Investig..

[130] Jin J., Zhong X.B. (2023). ASO drug Qalsody (tofersen) targets amyotrophic lateral sclerosis. Trends Pharmacol. Sci..

[131] Miller T., Cudkowicz M., Shaw P.J., Andersen P.M., Atassi N., Bucelli R.C., Genge A., Glass J., Ladha S., Ludolph A.L. (2020). Phase 1-2 trial of antisense oligonucleotide tofersen for SOD1 ALS. N. Engl. J. Med..

[132] Miller T.M., Cudkowicz M.E., Genge A., Shaw P.J., Sobue G., Bucelli R.C., Chiò A., Van Damme P., Ludolph A.C., Glass J.D. (2022). Trial of antisense oligonucleotide tofersen for SOD1 ALS. N. Engl. J. Med..

[133] Blair H.A. (2023). Tofersen: First approval. Drugs.

[134] Borel F., Gernoux G., Cardozo B., Metterville J.P., Cabrera G.T., Song L., Su Q., Gao G.P., Elmallah M.K., Brown R.H. (2016). Therapeutic rAAVrh10 mediated SOD1 silencing in adult SOD1(G93A) mice and nonhuman primates. Hum. Gene Ther..

[135] Arrowhead Pharmaceuticals Preclinical Profile of ARO-SOD1, an siRNA Therapy for SOD1-ALS [Poster]. https://ir.arrowheadpharma.com/static-files/804aa94c-7323-4917-a04f-bd0812d2e2fb.

[136] Mueller C., Berry J.D., McKenna-Yasek D.M., Gernoux G., Owegi M.A., Pothier L.M., Douthwright C.L., Gelevski D., Luppino S.D., Blackwood M. (2020). SOD1 suppression with adeno-associated virus and microRNA in familial ALS. N. Engl. J. Med..

[137] Ye J., Jiang L., Wang L., Pan Y., Wang X., Qu H., Liao X., Zhou X., Zhang S., Kang M. (2025). RAG-17, a novel siRNA therapy for SOD1-ALS: Safety and preliminary efficacy from a first-in-human trial (S5.003). Neurology.

[138] Assoni A.F., Foijer F., Zatz M. (2023). Amyotrophic lateral sclerosis, FUS and protein synthesis defects. Stem Cell Rev. Rep..

[139] Tarlarini C., Lunetta C., Mosca L., Avemaria F., Riva N., Mantero V., Maestri E., Quattrini A., Corbo M., Melazzini M.G. (2015). Novel FUS mutations identified through molecular screening in a large cohort of familial and sporadic amyotrophic lateral sclerosis. Eur. J. Neurol..

[140] Akiyama T., Warita H., Kato M., Nishiyama A., Izumi R., Ikeda C., Kamada M., Suzuki N., Aoki M. (2016). Genotype-phenotype relationships in familial amyotrophic lateral sclerosis with FUS/TLS mutations in Japan. Muscle Nerve.

[141] Vance C., Rogelj B., Hortobágyi T., De Vos K.J., Nishimura A.L., Sreedharan J., Hu X., Smith B., Ruddy D., Wright P. (2009). Mutations in FUS, an RNA processing protein, cause familial amyotrophic lateral sclerosis type 6. Science.

[142] Xiao X., Li M., Ye Z., He X., Wei J., Zha Y. (2024). FUS gene mutation in amyotrophic lateral sclerosis: A new case report and systematic review. Amyotroph. Lateral Scler. Front. Degener..

[143] Reber S., Stettler J., Filosa G., Colombo M., Jutzi D., Lenzken S.C., Schweingruber C., Bruggmann R., Bachi A., Barabino S.M. (2016). Minor intron splicing is regulated by FUS and affected by ALS-associated FUS mutants. EMBO J..

[144] Korobeynikov V.A., Lyashchenko A.K., Blanco-Redondo B., Jafar-Nejad P., Shneider N.A. (2022). Antisense oligonucleotide silencing of FUS expression as a therapeutic approach in amyotrophic lateral sclerosis. Nat. Med..

[145] Conte A., Lattante S., Zollino M., Marangi G., Luigetti M., Del Grande A., Servidei S., Trombetta F., Sabatelli M. (2012). P525L *FUS* mutation is consistently associated with a severe form of juvenile amyotrophic lateral sclerosis. Neuromuscul. Disord..

[146] Shneider N.A., Harms M.B., Korobeynikov V.A., Rifai O.M., Hoover B.N., Harrington E.A., Aziz-Zaman S., Singleton J., Jamil A., Madan V.R. (2025). Antisense oligonucleotide jacifusen for FUS-ALS: An investigator-initiated, multicentre, open-label case series. Lancet.

[147] Renton A.E., Majounie E., Waite A., Simon-Saánchez J., Rollinson S., Gibbs J.R., Schymick J.C., Laaksovirta H., van Swieten J.C., Myllykangas L. (2011). A hexanucleotide repeat expansion in C9ORF72 is the cause of chromosome 9p21-linked ALS-FTD. Neuron.

[148] Smeyers J., Banchi E.G., Latouche M. (2021). C9ORF72: What it is, what it does, and why it matters. Front. Cell. Neurosci..

[149] DeJesus-Hernandez M., Mackenzie I.R., Boeve B.F., Boxer A.L., Baker M., Rutherford N.J., Nicholson A.M., Finch N.A., Flynn H., Adamson J. (2011). Expanded GGGGCC hexanucleotide repeat in noncoding region of C9ORF72 causes chromosome 9p-linked FTD and ALS. Neuron.

[150] Majounie E., Renton A.E., Mok K., Dopper E.G., Waite A., Rollinson S., Chiò A., Restagno G., Nicolaou N., Simon-Sanchez J. (2012). Frequency of the C9orf72 hexanucleotide repeat expansion in patients with amyotrophic lateral sclerosis and frontotemporal dementia: A cross-sectional study. Lancet Neurol..

[151] Van Mossevelde S., van der Zee J., Cruts M., Van Broeckhoven C. (2017). Relationship between C9orf72 repeat size and clinical phenotype. Curr. Opin. Genet. Dev..

[152] Iacoangeli A., Al Khleifat A., Jones A.R., Sproviero W., Shatunov A., Opie-Martin S., Morrison K.E., Shaw P.J., Shaw C.E., Fogh I. (2019). C9orf72 intermediate expansions of 24–30 repeats are associated with ALS. Acta Neuropathol. Commun..

[153] Sellier C., Corcia P., Vourc’h P., Dupuis L. (2024). C9ORF72 hexanucleotide repeat expansion: From ALS and FTD to a broader pathogenic role?. Rev. Neurol..

[154] Braems E., Swinnen B., Van Den Bosch L. (2020). C9orf72 loss-of-function: A trivial, stand-alone or additive mechanism in C9 ALS/FTD?. Acta Neuropathol..

[155] Shi Y., Lin S., Staats K.A., Li Y., Chang W.-H., Hung S.-T., Hendricks E., Linares G.R., Wang Y., Son E.Y. (2018). Haploinsufficiency leads to neurodegeneration in C9ORF72 ALS/FTD human induced motor neurons. Nat. Med..

[156] Swinnen B., Robberecht W., Van Den Bosch L. (2020). RNA toxicity in non-coding repeat expansion disorders. EMBO J..

[157] Ash P.E.A., Bieniek K.F., Gendron T.F., Caulfield T., Lin W.-L., DeJesus-Hernandez M., van Blitterswijk M.M., Jansen-West K., Paul J.W., Rademakers R. (2013). Unconventional translation of C9ORF72 GGGGCC expansion generates insoluble polypeptides specific to c9FTD/ALS. Neuron.

[158] Lagier-Tourenne C., Baughn M., Rigo F., Sun S., Liu P., Li H.-R., Jiang J., Watt A.T., Chun S., Katz M. (2013). Targeted degradation of sense and antisense C9orf72 RNA foci as therapy for ALS and frontotemporal degeneration. Proc. Natl. Acad. Sci. USA.

[159] Tran H., Moazami M.P., Yang H., McKenna-Yasek D., Douthwright C.L., Pinto C., Metterville J., Shin M., Sanil N., Dooley C. (2022). Suppression of mutant C9orf72 expression by a potent mixed backbone antisense oligonucleotide. Nat. Med..

[160] Jiang J., Zhu Q., Gendron T.F., Saberi S., McAlonis-Downes M., Seelman A., Stauffer J.E., Jafar-Nejad P., Drenner K., Schulte D. (2016). Gain of toxicity from ALS/FTD-linked repeat expansions in C9ORF72 is alleviated by antisense oligonucleotides targeting GGGGCC-containing RNAs. Neuron.

[161] Berg L.H.v.D., Rothstein J.D., Shaw P.J., Babu S., Benatar M., Bucelli R.C., Genge A., Glass J.D., Hardiman O., Libri V. (2024). Safety, tolerability, and pharmacokinetics of antisense oligonucleotide BIIB078 in adults with C9orf72-associated amyotrophic lateral sclerosis: A phase 1, randomised, double-blind, placebo-controlled, multiple ascending dose study. Lancet Neurol..

[162] Meijboom K.E., Brown R.H. (2022). Approaches to gene modulation therapy for ALS. Neurotherapeutics.

[163] Ratti A., Buratti E. (2016). Physiological functions and pathobiology of TDP-43 and FUS/TLS proteins. J. Neurochem..

[164] Guo L., Shorter J. (2017). Biology and pathobiology of TDP-43 and emergent therapeutic strategies. Cold Spring Harb. Perspect. Med..

[165] Giordana M.T., Piccinini M., Grifoni S., De Marco G., Vercellino M., Magistrello M., Pellerino A., Buccinnà B., Lupino E., Rinaudo M.T. (2010). TDP-43 redistribution is an early event in sporadic amyotrophic lateral sclerosis. Brain Pathol..

[166] Suk T.R., Rousseaux M.W.C. (2020). The role of TDP-43 mislocalization in amyotrophic lateral sclerosis. Mol. Neurodegener..

[167] Kabashi E., Lin L., Tradewell M.L., Dion P.A., Bercier V., Bourgouin P., Rochefort D., Hadj S.B., Durham H.D., Velde C.V. (2010). Gain and loss of function of ALS-related mutations of TARDBP (TDP-43) cause motor deficits in vivo. Hum. Mol. Genet..

[168] Babazadeh A., Rayner S.L., Lee A., Chung R.S. (2023). TDP-43 as a therapeutic target in neurodegenerative diseases: Focusing on motor neuron disease and frontotemporal dementia. Ageing Res. Rev..

[169] Takeuchi T., Maeta K., Ding X., Oe Y., Takeda A., Inoue M., Nagano S., Fujihara T., Matsuda S., Ishigaki S. (2023). Sustained therapeutic benefits by transient reduction of TDP-43 using ENA-modified antisense oligonucleotides in ALS/FTD mice. Mol. Ther. Nucleic Acids.

[170] Becker L.A., Huang B., Bieri G., Ma R., Knowles D.A., Jafar-Nejad P., Messing J., Kim H.J., Soriano A., Auburger G. (2017). Therapeutic reduction of ataxin-2 extends lifespan and reduces pathology in TDP-43 mice. Nature.

[171] Amado D.A., Robbins A.B., Smith A.R., Whiteman K.R., Chillon Bosch G., Chen Y., Fuller J.A., Izda A., Nelson S., Dichter A.I. (2024). AAV-based delivery of RNAi targeting Ataxin-2 improves survival, strength, and pathology in mouse models of rapidly and slowly progressive sporadic ALS. bioRxiv.

[172] Biogen and Ionis Pharmaceuticals Biogen and Ionis Announce Topline Phase 1/2 Study Results of Investigational Drug in Amyotrophic Lateral Sclerosis. https://investors.biogen.com/news-releases/news-release-details/biogen-and-ionis-announce-topline-phase-12-study-results.

[173] Melamed Z., López-Erauskin J., Baughn M.W., Zhang O., Drenner K., Sun Y., Freyermuth F., McMahon M.A., Beccari M.S., Artates J.W. (2019). Premature polyadenylation-mediated loss of stathmin-2 is a hallmark of TDP-43-dependent neurodegeneration. Nat. Neurosci..

[174] Klim J.R., Williams L.A., Limone F., San Juan I.G., Davis-Dusenbery B.N., Mordes D.A., Burberry A., Steinbaugh M.J., Gamage K.K., Kirchner R. (2019). ALS-implicated protein TDP-43 sustains levels of STMN2, a mediator of motor neuron growth and repair. Nat. Neurosci..

[175] Shin J.E., Geisler S., DiAntonio A. (2014). Dynamic regulation of SCG10 in regenerating axons after injury. Exp. Neurol..

[176] Baughn M.W., Melamed Z., López-Erauskin J., Beccari M.S., Ling K., Zuberi A., Presa M., Gonzalo-Gil E., Maimon R., Vazquez-Sanchez S. (2023). Mechanism of STMN2 cryptic splice-polyadenylation and its correction for TDP-43 proteinopathies. Science.

[177] Genge A., Salmon K., Polzer J., Martinez C., Boggs B., Eon V., Ganti R., Hinckley S., Johnson K., Elbaum D. (2024). QRL-201-01—A multi-center, randomized, double-blind, placebo-controlled multiple ascending dose study to evaluate the safety and tolerability of QRL-201 in amyotrophic lateral sclerosis (P6-11.010). Neurology.

[178] Phares S., Phillip K., Trusheim M. (2025). Clinical development success rates for durable cell and gene therapies. Nat. Rev. Drug Discov..

[179] Sumner C.J., Crawford T.O. (2022). Early treatment is a lifeline for infants with SMA. Nat. Med..

[180] Rothgangl T., Dennis M.K., Lin P.J.C., Oka R., Witzigmann D., Villiger L., Qi W., Hruzova M., Kissling L., Lenggenhager D. (2021). In vivo adenine base editing of PCSK9 in macaques reduces LDL cholesterol levels. Nat. Biotechnol..

[181] Janssen P., Isa T., Lanciego J., Leech K., Logothetis N., Poo M.-M., Mitchell A.S. (2023). Visualizing advances in the future of primate neuroscience research. Curr. Res. Neurobiol..

[182] Westerling-Bui A.D., Fast E.M., Soare T.W., Venkatachalan S., DeRan M., Fanelli A.B., Kyrychenko S., Hoang H., Corriea G.M., Zhang W. (2022). Transplanted organoids empower human preclinical assessment of drug candidate for the clinic. Sci. Adv..

[183] Evangelisti C., Ramadan S., Orlacchio A., Panza E. (2024). Experimental cell models for investigating neurodegenerative diseases. Int. J. Mol. Sci..

[184] Fernandopulle M.S., Prestil R., Grunseich C., Wang C., Gan L., Ward M.E. (2018). Transcription factor-mediated differentiation of human iPSCs into neurons. Curr. Protoc. Cell Biol..

[185] Giacomelli E., Vahsen B.F., Calder E.L., Xu Y., Scaber J., Gray E., Dafinca R., Talbot K., Studer L. (2022). Human stem cell models of neurodegeneration: From basic science of amyotrophic lateral sclerosis to clinical translation. Cell Stem Cell.

[186] Answer ALS. https://www.answerals.org.

[187] Okano H., Morimoto S. (2022). iPSC-based disease modeling and drug discovery in cardinal neurodegenerative disorders. Cell Stem Cell.

[188] Grass T., Dokuzluoglu Z., Buchner F., Rosignol I., Thomas J., Caldarelli A., Dalinskaya A., Becker J., Rost F., Marass M. (2024). Isogenic patient-derived organoids reveal early neurodevelopmental defects in spinal muscular atrophy initiation. Cell Rep. Med..

[189] Faravelli I., Rinchetti P., Tambalo M., Simutin I., Mapelli L., Mancinelli S., Miotto M., Rizzuti M., D’aNgelo A., Cordiglieri C. (2025). Targeted antisense oligonucleotide treatment rescues developmental alterations in spinal muscular atrophy organoids. bioRxiv.

[190] Szebényi K., Wenger L.M.D., Sun Y., Dunn A.W.E., Limegrover C.A., Gibbons G.M., Conci E., Paulsen O., Mierau S.B., Balmus G. (2021). Human ALS/FTD brain organoid slice cultures display distinct early astrocyte and targetable neuronal pathology. Nat. Neurosci..

[191] Gao C., Shi Q., Pan X., Chen J., Zhang Y., Lang J., Wen S., Liu X., Cheng T.-L., Lei K. (2024). Neuromuscular organoids model spinal neuromuscular pathologies in C9orf72 amyotrophic lateral sclerosis. Cell Rep..

[192] van der Geest A.T., Jakobs C.E., Ljubikj T., Huffels C.F.M., Luna M.C., de Sá R.V., Adolfs Y., de Wit M., Rutten D.H., Kaal M. (2024). Molecular pathology, developmental changes and synaptic dysfunction in (pre-)symptomatic human C9ORF72-ALS/FTD cerebral organoids. Acta Neuropathol. Commun..

[193] U.S. Food and Drug Administration Rare Diseases: Natural History Studies for Drug Development. Draft Guidance for Industry; FDA-2019-D-0481. https://www.fda.gov/regulatory-information/search-fda-guidance-documents/rare-diseases-natural-history-studies-drug-development.

[194] Alqahtani A., Kokkinis A., Zizzi C., Dilek N., Fischbeck K.H., Heatwole C.R., Grunseich C. (2023). Patient-reported impact of symptoms in spinal and bulbar muscular atrophy. Neurol. Clin. Pract..

[195] Grunseich C., Patankar A., Amaya J., Watts J.A., Li D., Ramirez P., Schindler A.B., Fischbeck K.H., Cheung V.G. (2020). Clinical and molecular aspects of senataxin mutations in amyotrophic lateral sclerosis 4. Ann. Neurol..

[196] FDA-NIH Biomarker Working Group (2017). BEST (Biomarkers, EndpointS, and Other Tools) Resource.

[197] Klickovic U., Zampedri L., Sinclair C.D., Wastling S.J., Trimmel K., Howard R.S., Malaspina A., Sharma N., Sidle K., Emira A. (2019). Skeletal muscle MRI differentiates SBMA and ALS and correlates with disease severity. Neurology.

[198] Tebbenkamp A.T., Huggett S.B., Lombardi V., Zampedri L., AlQahtani A., Kokkinis A., Malaspina A., Rinaldi C., Grunseich C., Fratta P. (2024). Protein biomarker signature in patients with spinal and bulbar muscular atrophy. JCI Insight.

[199] Xing X., Liu X., Li X., Li M., Wu X., Huang X., Xu A., Liu Y., Zhang J. (2025). Insights into spinal muscular atrophy from molecular biomarkers. Neural Regen. Res..

[200] Arbab M., Matuszek Z., Kray K.M., Du A., Newby G.A., Blatnik A.J., Raguram A., Richter M.F., Zhao K.T., Levy J.M. (2023). Base editing rescue of spinal muscular atrophy in cells and in mice. Science.

[201] Alves C.R.R., Ha L.L., Yaworski R., Sutton E.R., Lazzarotto C.R., Christie K.A., Reilly A., Beauvais A., Doll R.M., de la Cruz D. (2024). Optimization of base editors for the functional correction of SMN2 as a treatment for spinal muscular atrophy. Nat. Biomed. Eng..

[202] Kempthorne L., Vaizoglu D., Cammack A.J., Carcolé M., Roberts M.J., Mikheenko A., Fisher A., Suklai P., Muralidharan B., Kroll F. (2025). Dual-targeting CRISPR-CasRx reduces C9orf72 ALS/FTD sense and antisense repeat RNAs in vitro and in vivo. Nat. Commun..

[203] McCallister T.X., Lim C.K.W., Singh M., Zhang S., Ahsan N.S., Terpstra W.M., Xiong A.Y., Zeballos C.M.A., Powell J.E., Drnevich J. (2025). A high-fidelity CRISPR-Cas13 system improves abnormalities associated with C9ORF72-linked ALS/FTD. Nat. Commun..

[204] Bairqdar A., Karitskaya P.E., Stepanov G.A. (2024). Expanding Horizons of CRISPR/Cas Technology: Clinical Advancements, Therapeutic Applications, and Challenges in Gene Therapy. Int. J. Mol. Sci..

[205] Wang Z., Huang J., Yun D. (2026). Current and Emerging Therapeutic Strategies for Amyotrophic Lateral Sclerosis: From Pharmacological Approaches to Gene and Stem Cell Therapies. Front. Neurol..

[206] Maragakis N.J., de Carvalho M., Weiss M.D. (2023). Therapeutic Targeting of ALS Pathways: Refocusing an Incomplete Picture. Ann. Clin. Transl. Neurol..

[207] Ottoboni L., Panicucci C., Magni G., Gagliardi D., Ripolone M., Napoli L., Moggio M., Comi G.P., Bruno C., Corti S.P. (2026). Skeletal Muscle in Spinal Muscular Atrophy: Critical Insights from Pathogenesis to Therapeutic Strategies. Neurobiol. Dis..

[208] D’sOuza A., Nozohouri S., Bleier B.S., Amiji M.M. (2023). CNS Delivery of Nucleic Acid Therapeutics: Beyond the Blood–Brain Barrier and Towards Specific Cellular Targeting. Pharm. Res..

